# *In vivo* cerebellar circuit function is disrupted in an *mdx* mouse model of Duchenne muscular dystrophy

**DOI:** 10.1242/dmm.040840

**Published:** 2019-12-09

**Authors:** Trace L. Stay, Lauren N. Miterko, Marife Arancillo, Tao Lin, Roy V. Sillitoe

**Affiliations:** 1 Department of Pathology and Immunology, Baylor College of Medicine, Houston, TX 77030, USA; 2 Department of Neuroscience, Baylor College of Medicine, Houston, TX 77030, USA; 3 Jan and Dan Duncan Neurological Research Institute of Texas Children's Hospital, 1250 Moursund Street, Suite 1325, Houston, TX 77030, USA; 4 Program in Developmental Biology, Baylor College of Medicine, Houston, TX 77030, USA

**Keywords:** Duchenne muscular dystrophy, *mdx* mice, Cerebellum, Purkinje cell, Cerebellar nuclei, Circuitry, *In vivo* electrophysiology

## Abstract

Duchenne muscular dystrophy (DMD) is a debilitating and ultimately lethal disease involving progressive muscle degeneration and neurological dysfunction. DMD is caused by mutations in the dystrophin gene, which result in extremely low or total loss of dystrophin protein expression. In the brain, dystrophin is heavily localized to cerebellar Purkinje cells, which control motor and non-motor functions. *In vitro* experiments in mouse Purkinje cells revealed that loss of dystrophin leads to low firing rates and high spiking variability. However, it is still unclear how the loss of dystrophin affects cerebellar function in the intact brain. Here, we used *in vivo* electrophysiology to record Purkinje cells and cerebellar nuclear neurons in awake and anesthetized female *mdx* (also known as *Dmd*) mice. Purkinje cell simple spike firing rate is significantly lower in *mdx* mice compared to controls. Although simple spike firing regularity is not affected, complex spike regularity is increased in *mdx* mutants. Mean firing rate in cerebellar nuclear neurons is not altered in *mdx* mice, but their local firing pattern is irregular. Based on the relatively well-preserved cytoarchitecture in the *mdx* cerebellum, our data suggest that faulty signals across the circuit between Purkinje cells and cerebellar nuclei drive the abnormal firing activity. The *in vivo* requirements of dystrophin during cerebellar circuit communication could help explain the motor and cognitive anomalies seen in individuals with DMD.

This article has an associated First Person interview with the first author of the paper.

## Abstract (precis)

**Summary:** The *mdx* mouse model of Duchenne muscular dystrophy (DMD) has *in vivo* abnormalities in cerebellar spike firing, which could contribute to the neurological symptoms observed in individuals with DMD.

## INTRODUCTION

Duchenne muscular dystrophy (DMD) is a devastating X-linked disease that affects ∼1 in 5000 boys (Guiraud et al., 2015). DMD is caused by mutations in the dystrophin gene (*DMD*) (Burghes et al., 1987; Hoffman et al., 1987; Monaco et al., 1986). The mutations eliminate the expression of the 427 kDa protein dystrophin, or lower it to less than 5% of normal. *DMD* mutations also cause the milder disease, Becker muscular dystrophy, as well as X-linked dilated cardiomyopathy. Interestingly, although heterozygous female carriers of *DMD* mutations are typically asymptomatic, up to ∼8% of these carriers are considered as manifesting carriers, who develop symptoms ranging from mild muscle weakness to a rapidly progressive DMD-like muscular dystrophy (Birnkrant et al., 2018; Moser and Emery, 1974; Norman and Harper, 1989; Taylor et al., 2007). Female carriers have also been reported to have cognitive abnormalities (Imbornoni et al., 2014; Mercier et al., 2013; Papa et al., 2016). Dystrophin functions as a tether for stabilizing protein complexes, and, in the brain, it also interacts with membrane proteins that mediate neuronal communication (Pilgram et al., 2010; Waite et al., 2009). Accordingly, loss of dystrophin can impair brain function (Anderson et al., 2002; Mirski and Crawford, 2014; Pereira da Silva et al., 2018; Snow et al., 2014). Cognition and movement are often affected, although the neural bases of these behavioral defects are unclear. In this study, we sought to gain a deeper understanding of how neuronal signals are altered in the DMD brain. Towards this, we used an *mdx* (also known as *Dmd*) mouse model to test how the loss of dystrophin (Dp427 isoform) alters cerebellar function by measuring neuronal activity *in vivo* (Grady et al., 2006; Ryder-Cook et al., 1988; Sicinski et al., 1989; Sillitoe et al., 2003).

Dystrophin protein complexes are heavily expressed in the cerebellum, where they are localized predominantly to Purkinje cells (Blake et al., 1999; Lidov et al., 1990, 1993; Sillitoe et al., 2003). Purkinje cells are the principal cell type of the cerebellum and the computational center for executing all cerebellar-dependent behaviors (Reeber et al., 2013). In mice, the loss of dystrophin dramatically alters Purkinje cell microcircuit organization (Sillitoe et al., 2003). Such structural alterations are consistent with the abnormal behaviors in *mdx* mutant mice, including uncoordinated movement (Grady et al., 2006). These molecular and behavioral defects are also consistent with defects in neuronal activity. *In vitro* electrophysiology experiments demonstrated that dissociated Purkinje cell firing activity is compromised in *mdx* mice (Snow et al., 2014). They found that Purkinje cells from the *mdx* mice fired more irregularly than those from control mice and that the membrane potential was hyperpolarized (Snow et al., 2014). The authors also reported a lower-than-normal Purkinje cell firing frequency in dissociated *mdx* Purkinje cells. Their results are consistent with other reports that showed a reduction in the number of GABA synapses on Purkinje cells (Kueh et al., 2011), aberrant GABA release and uptake in cerebellar synaptosomes (Pereira da Silva et al., 2018), and a reduction in postsynaptic long-term depression (LTD) in *mdx* Purkinje cells [although Sesay et al. (1996) found no LTP changes in *mdx* urethane-anesthetized hippocampal cells, which could be related to the anesthetic effects of urethane (Hara and Harris, 2002)]. Interestingly, homosynaptic LTD at parallel fiber–Purkinje cell synapses is enhanced in *mdx* mutant mice (Anderson et al., 2010), but how altering these intrinsic membrane properties of Purkinje cells affects circuit function in the intact cerebellum *in vivo* remains largely unsolved.

The cerebellum controls a variety of motor behaviors, including coordination, learning, balance and posture (Reeber et al., 2013; Thach, 2014). It may also play a pivotal role in non-motor functions such as cognition, language, emotion, reward, social interactions and spatial working memory (Buckner, 2013; Carta et al., 2019; D'Angelo and Casali, 2013; McAfee et al., 2019; Stoodley, 2012). The execution of all these behaviors requires proper Purkinje cell function (Heiney et al., 2014; Tsai et al., 2012; White et al., 2014). Importantly, motor and non-motor Purkinje cell functions could be relevant to DMD (Anderson et al., 2002). In either case, the canonical Purkinje cell circuit is likely involved. Purkinje cells receive direct excitatory input from climbing fibers and granule cell parallel fiber axons, and inhibitory inputs from stellate and basket cell interneurons. Purkinje cells are the only output of the cerebellar cortex, and inhibit neurons in the cerebellar and vestibular nuclei. The cerebellar nuclei are located in the inner core of the cerebellum and provide the main output of the cerebellum. We hypothesize that, in DMD, Purkinje cells fire abnormally and, as a consequence, behavioral output is negatively impacted. In this study, we address how the loss of dystrophin affects Purkinje cell function and specifically assess how Purkinje cell firing activity is affected *in vivo* in mice (Arancillo et al., 2015; White et al., 2014).

We performed *in vivo* extracellular recordings in both anesthetized and awake adult control and *mdx* mutant mice. The main objective of this study was to investigate whether functional changes occur *in vivo* in Purkinje cells and in their downstream target cerebellar nuclear cells when dystrophin is absent. Our experiments revealed three main findings: (1) Purkinje cell simple spike firing rate is significantly lower in *mdx* mutant mice than in controls; (2) the pattern of firing in *mdx* cerebellar nuclear neurons is more irregular than that in controls; and (3) the overall firing features of the *mdx* Purkinje cells are highly reminiscent of the defects observed in several mutant mouse strains that model cerebellar disease, including ataxia and dystonia (Fremont et al., 2014; Gao et al., 2012; White and Sillitoe, 2017; White et al., 2014, 2016a). Altogether, our findings provide a functional correlate to the well-studied anatomical and molecular pathogenesis of DMD (Guiraud and Davies, 2019), and provide a valuable set of cell activity data for evaluating exactly how different brain functions might be affected *in vivo* in the different muscular dystrophies.

## RESULTS

### Circuit architecture is unaltered in *mdx* mice

**Fig. 1.**
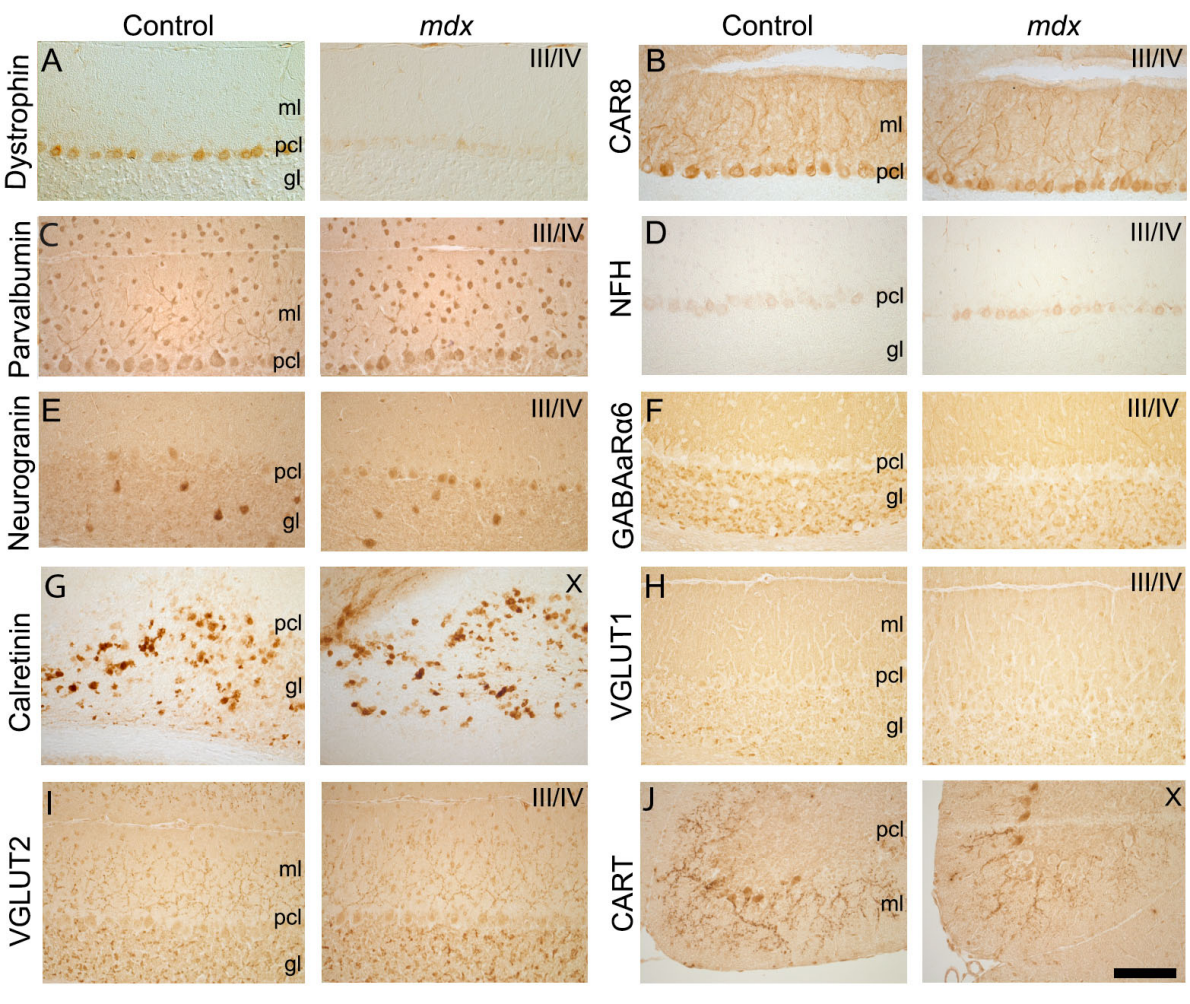
**The molecular expression profile of cerebellar neurons is similar in *mdx* and control mice.** (A) Control mice express dystrophin in Purkinje cells, whereas *mdx* mice do not. (B-J) Control and *mdx* mice have similar expression patterns for markers of Purkinje cells (B), inhibitory neurons (C-E), and excitatory cells and inputs (F-J). (B) CAR8 labels Purkinje cell somata and dendrites (axons are also labeled with CAR8 but are not seen in this tissue orientation). (C) Parvalbumin (PV) labels inhibitory Purkinje cells, molecular layer interneurons and granular layer interneurons. (D) Neurofilament heavy (NFH) labels Purkinje cells and basket cell axons. (E) Neurogranin labels Golgi cells in the adult mouse. (F) Gamma-amino-butyric-acid receptor alpha 6 (GABAaRα6) labels granule cells and their parallel fiber axons. (G) Calretinin labels a subset of unipolar brush cells. (H) Vesicular glutamate transporter 1 (VGLUT1) labels parallel fiber and mossy fiber terminals. (I) Vesicular glutamate transporter 2 (VGLUT2) labels climbing fiber and mossy fiber terminals. (J) Cocaine- and amphetamine-related transcript peptide (CART) labels climbing fiber axons and terminals mainly in lobules IX and X. Scale bar: 200 µm. gl, granular layer; ml, molecular layer; pcl, Purkinje cell layer. *N*=3 mice of each genotype.

Mouse models of disease often exhibit changes in the anatomy of cerebellar microcircuits (Cendelin, 2014; Lalonde and Strazielle, 2007; Landis and Landis, 1978), which could affect the normal properties of cerebellar function (Barmack and Yakhnitsa, 2008; Davie et al., 2008; De Zeeuw et al., 2011; Ruigrok et al., 2011; Schmolesky et al., 2002). To address whether this is the case in C57BL/10ScSn-*Dmd^mdx^*/J homozygous mutant mice (hereafter referred to as *mdx* mice), we examined the regional localization and layered tri-laminar distribution of cerebellar cells in *mdx* mice by immunostaining with a panel of cell-specific molecular markers (Fig. 1). As expected from this mouse model, we confirmed that the *mdx* Purkinje cells lack the dystrophin protein (Dp427; Fig. 1A). We first examined the inhibitory neurons of the cerebellar cortex using markers that identify each cell type (Reeber and Sillitoe, 2011; White et al., 2014, 2016b). Using CAR8 staining (Miterko et al., 2019a; White et al., 2016a), we found that mutant Purkinje cells were arranged into the characteristic monolayer and exhibited their signature, wide-spanning dendritic architecture, as also observed in C57BL/10ScSnJ controls (hereafter referred to as controls; Fig. 1B). Overall inhibitory interneuron distribution was also similar between genotypes when revealed with parvalbumin staining (Fig. 1C). Basket cell projections were identified in the *mdx* mice, as seen through staining for NFH (also known as Nefh) (Fig. 1D). Additionally, the overall distribution of Golgi cells in the granule cell layer of *mdx* mice was equivalent to that in controls, as shown by neurogranin staining (Fig. 1E). These data suggest that inhibitory neurons in the cerebellar cortex of *mdx* mice have similar dorsoventral patterning and regional distributions to those in the same region in control mice.

We next examined the excitatory neurons and the excitatory afferent inputs to the cerebellar cortex through immunohistochemistry (White et al., 2014, 2016b). Adult differentiated granule cells were stained with GABAaRα6; they were correctly accumulated in the granule cell layer in *mdx* mice (Fig. 1F). Unipolar brush cells are a specialized type of neuron localized predominantly to lobules IX and X (Diño et al., 1999), with a secondary site of localization in lobules VI and VII (Sillitoe et al., 2003). In *mdx* mice, the unipolar brush cells were heavily immunoreactive for calretinin, and, as in control mice, they were located mainly in lobules IX and X (Fig. 1G). We used VGLUT1 (also known as Slc17a7) expression to demonstrate that the terminals of granule cell projections, found on their parallel fibers, were represented in their typical abundance in the molecular layer in *mdx* mice (Fig. 1H). Mossy fiber inputs to the granular layer and climbing fiber inputs to the molecular layer were clear in *mdx* mice, with both classes of terminals revealed by VGLUT2 (also known as Slc17a6) (Fig. 1I). The climbing fibers were also visualized with CART (encoded by the *Cartpt* gene) (Fig. 1J), which showed the normal climbing trajectory of axons in the molecular layer of *mdx* mice. Overall, the normal cellular layering, cell distribution, neuronal patterning and afferent terminal field localization in the cerebellar circuit of *mdx* mice argues against any major developmental morphogenetic rearrangements as a major contributor to the neurological deficits observed in the *mdx* mice.

### Lack of overt neurodegeneration in *mdx* mice

**Fig. 2.**
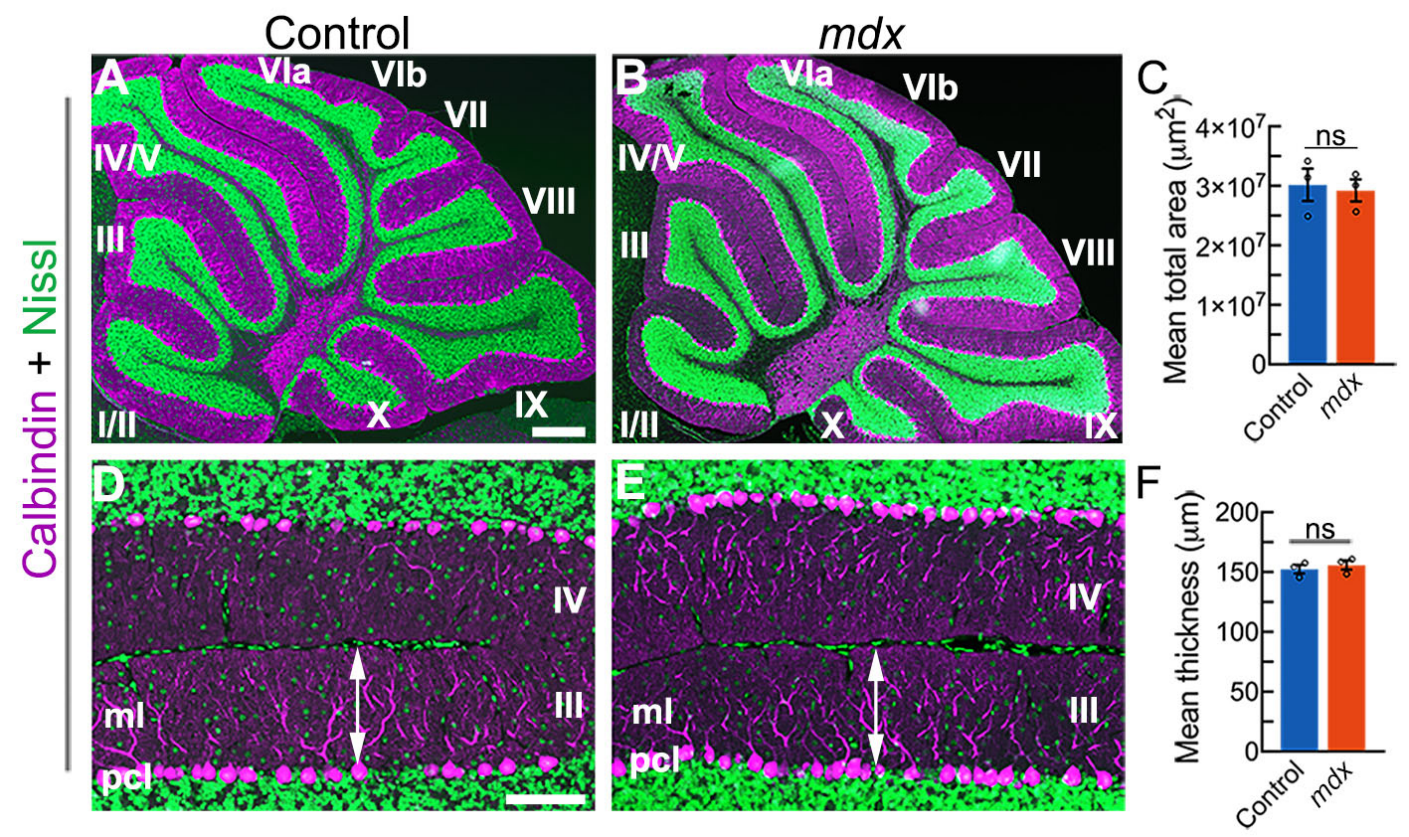
**Gross cerebellar morphology does not differ between *mdx* mice and controls.** (A,B) Representative mid-sagittal sections showing Purkinje cells stained with calbindin (magenta) and granule cells stained with fluorescent Nissl (green). (C) No significant difference in cerebellar size was found between genotypes [control=30.1×10^6^±2.8×10^6^ μm^2^, *mdx=*29.2×10^6^±1.9×10^6^ μm^2^, two-tailed Student's *t*-test, *N*=3 mice of each genotype, *n*=3-8 sections per animal, t(4)=0.280, *P*=0.79]. (D,E) Higher-power images from lobules III-IV illustrate how molecular layer thickness was measured perpendicular to the Purkinje cell monolayer adjacent to the primary fissure. (F) No significant difference in molecular layer thickness was found between genotypes [control mean=152.3±3.7 μm, *mdx* mean=155.7±4.0 μm, two-tailed Student's *t*-test, *N*=3 mice of each genotype, *n*=4-8 sections per animal, t(4)=−0.623, *P*=0.57]. Scale bars: 500 µm (A); 50 µm (D). ml, molecular layer; ns, non-significant; pcl, Purkinje cell layer.

Cerebellar size and molecular layer thickness can also be altered in disease models, with variations from the normal range indicative of improper development and function (Hansen et al., 2013). These two measures are also useful anatomical readouts of neurodegeneration and cell loss. If Purkinje cells succumb to degeneration, regression of their large dendritic trees, the most notable entity of the molecular layer, results in a significant decrease in the size of the cerebellar cortex (Miterko et al., 2019a). Towards this, we analyzed cerebellar size and molecular layer thickness in *mdx* and control mice to provide an overall impression of cerebellar architectural integrity. The distance we measured was from the apical edge of the Purkinje cell soma to the pial boundary of the molecular layer region directly above (Brown et al., 2019; White et al., 2014, 2016a). Measurements from three mice of each genotype did not reveal a significant difference in total cerebellar size or molecular layer thickness [Fig. 2; cerebellar size: control=30.1×10^6^±2.8×10^6^ μm^2^, *mdx=*29.2×10^6^±1.9×10^6^ μm^2^, t(4)=0.280, *P*=0.79; molecular layer thickness: control=152.3±3.7 μm, *mdx=*155.7±4.0 μm, t(4)=−0.623, *P*=0.57]. A more detailed analysis of Purkinje cell numbers, cerebellar nuclear areas and cerebellar nuclear densities additionally confirmed that overt neurodegeneration does not occur in *mdx* mice at the ages examined (Fig. S1). The number of Purkinje cells in *mdx* mice does not differ from that in controls when counted from all vermis lobules or when counts are taken from specific zones [Fig. S1A-D; control=569.2±32.7 (whole), 256.0±12.9 (anterior), 169.4±7.4 (central), 143.8±14.3 (posterior/nodular), *mdx=*578.7±6.7 (whole), 260.6±3.6 (anterior), 181.4±5.1 (central), 136.7±2.3 (posterior/nodular); t(4)=−0.286, *P*=0.79, t(4)=−0.340, *P*=0.75, t(4)=−1.345, *P*=0.25, t(4)=0.489, *P*=0.65, respectively]. Likewise, the areas and densities of the cerebellar nuclei in the *mdx* mice are comparable to those of control mice [Fig. S1E-J; areas: control=1242.5±154.9 μm^2^ (fastigial, FN), 4051.9±385.4 μm^2^ (interposed, IN), *mdx=*1483.5±287.7 μm^2^ (FN), 3015.8±567.9 μm^2^ (IN); densities (number of nuclei/50 μm): control=17.36±1.61 (FN), 36.11±3.12 (IN), *mdx=*21.26±1.20 (FN), 33.19±1.14 (IN); t(4)=−0.737, *P*=0.50, t(4)=1.510, *P*=0.21, t(4)=−1.9646, *P*=0.12, t(4)=0.880, *P*=0.43, respectively]. These data suggest that the main cellular components of the cerebellar circuit maintain their anatomical integrity in the *mdx* mice.

### *In vivo* Purkinje cell function is abnormal in *mdx* mice

**Fig. 3.**
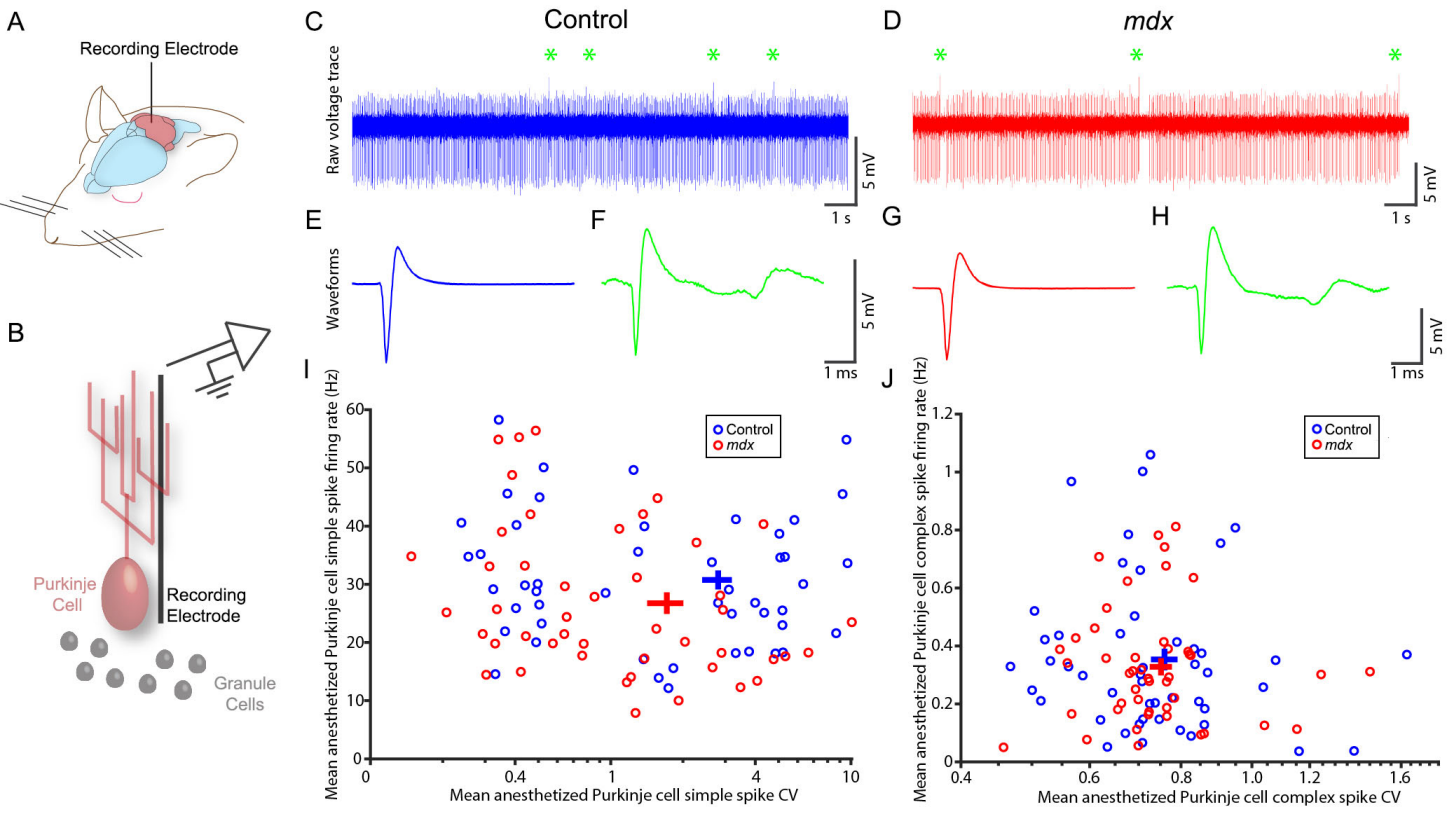
**Extracellular spontaneous firing properties of Purkinje cells in ketamine–dexmedetomidine-anesthetized *mdx* and control mice.** (A) Schematic showing the recording approach. (B) Schematic illustrating the electrode placement for targeting the Purkinje cells. (C) Example raw voltage trace from a control Purkinje cell, with complex spikes indicated by green asterisks. (D) As in C, for *mdx* mice. (E) Average simple spike waveform for the cell shown in C. (F) Average complex spike waveform for the cell in C. (G,H) Simple spike and complex spike waveforms for the *mdx* cell shown in D. (I) Mean simple spike firing rate (*y*-axis) was not significantly lower in *mdx* mice (red) compared to control mice (blue) when corrected for multiple comparisons. No statistically significant difference in mean coefficient of variance (CV) (*x*-axis) was found, although there was a trend toward lower variability in the *mdx* mice. (J) No significant differences in mean firing rate or mean CV for complex spikes were found. Circles are individual data points; crosses represent mean±s.e.m. *n*=48 control cells, *n*=46 *mdx* cells, *N*=12 mice of each genotype. Please refer to Table S1 for a list of the statistical tests used in this figure.

Having established that the gross anatomy and basic circuit features of the cerebellum are undisturbed in the *mdx* mice, we next tested functional properties (Fig. 3). We first recorded from anesthetized control and *mdx* Purkinje cells (Fig. 3A,B) to provide a large cell yield. Recordings revealed similar membrane voltages between the genotypes (e.g. example voltage traces in Fig. 3C,D), and, based on their firing signatures, both genotypes had recognizable Purkinje cells, with clear complex spikes confirming their cell identity (indicated by green asterisks in Fig. 3C,D). Additionally, individual simple spike and complex spike waveforms were comparable between control and *mdx* mice (Fig. 3E-H). These data provided us with confidence that if Purkinje cells in the *mdx* mice are functionally abnormal, it is due to changes in their spike firing patterns rather than to a change in or loss of the different spike types themselves [e.g. after conditional deletion of complex spike activity (White and Sillitoe, 2017)]. Therefore, in order to examine how the Purkinje cells behave *in vivo*, we analyzed and compared their mean firing summary statistics.

**Fig. 4.**
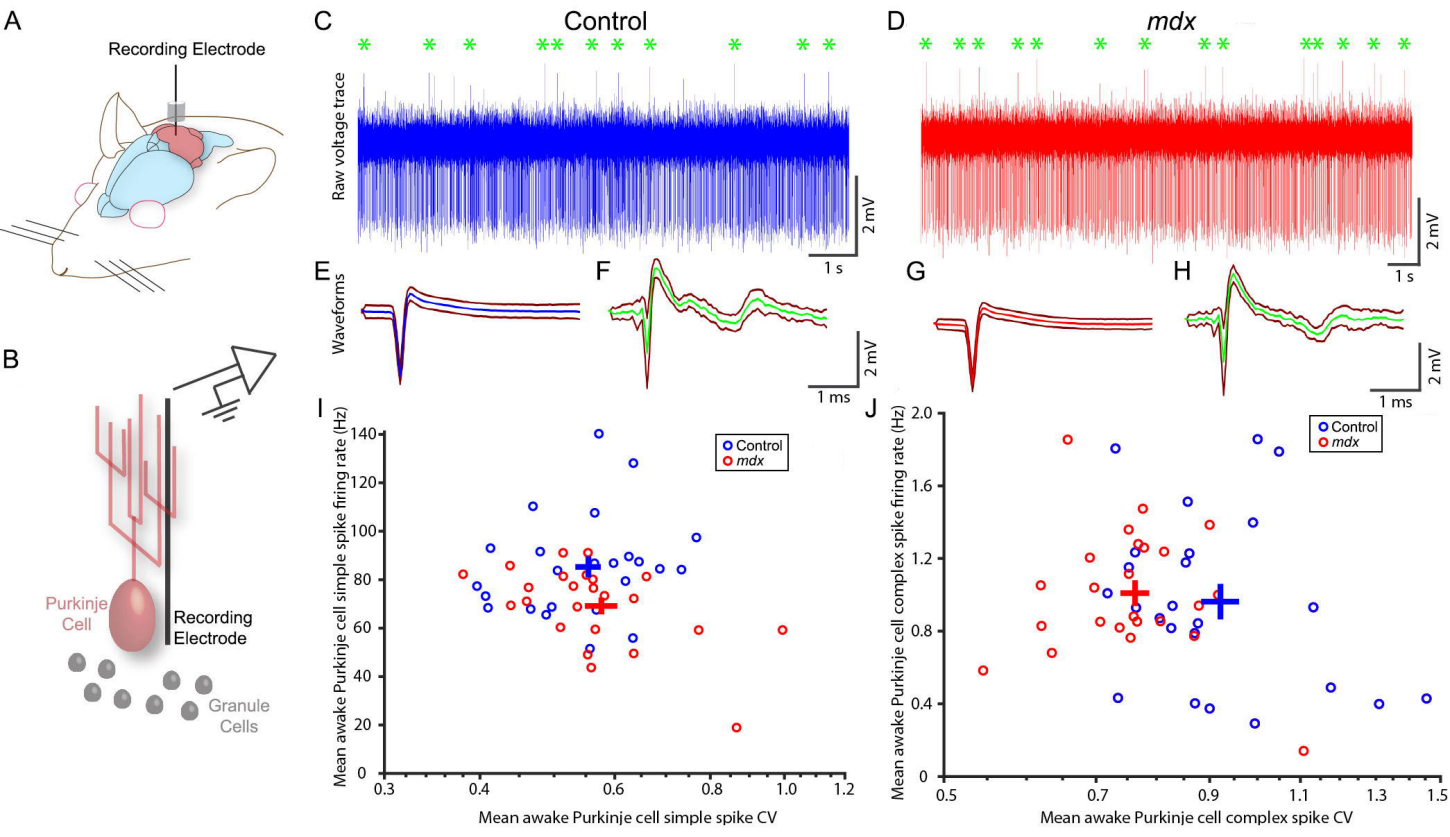
**Awake extracellular spontaneous firing properties of Purkinje cells in *mdx* and control mice.** (A) Schematic showing the recording approach. (B) Schematic illustrating the electrode placement for targeting the Purkinje cells. (C) Example raw voltage trace from a control Purkinje cell, with complex spikes indicated by green asterisks. (D) As in C, for *mdx* mice. (E) Average simple spike waveform for the cell shown in C. (F) Average complex spike waveform for the cell in C. (G,H) Simple spike and complex spike waveforms for the *mdx* cell shown in D. (I) Mean simple spike firing rate (*y*-axis) was significantly lower in *mdx* mice (red) compared to controls (blue). No statistically significant difference in mean CV (*x*-axis) was found. (J) Complex spikes in *mdx* mice have significantly lower firing variability compared to those in controls, but firing rates do not differ between the genotypes. Circles are individual data points; crosses represent mean±s.e.m. *n*=24 cells of each genotype, *N*=4 mice of each genotype. Please refer to Table S1 for a list of the statistical tests used in this figure.

Genotype had a significant effect on the combined set of anesthetized simple spike mean firing rate, mean coefficient of variance (CV) and mean coefficient of variance of adjacent interspike intervals (CV2) [one-way MANOVA, Wilk's λ(1,92)=0.9028, *P*=0.026]. However, even though simple spike firing was ∼13% lower in *mdx* animals compared to controls (Fig. 3I; 26.8±1.9 Hz vs 30.8±1.6 Hz; Wilcoxon rank sum test, z=1.99, *P*=0.047), this change was not significant when corrected for multiple comparisons. Neither overall irregularity (CV) nor local irregularity (CV2) showed significant differences (z=1.76, *P*=0.079 and z=−1.48, *P*=0.14, respectively). Genotype also did not have a significant effect on complex spike firing [Fig. 3J; Wilk's λ(1,92)=0.9929, *P*=0.89]. Given the trend toward lower simple spike firing in *mdx* mice, our previous data demonstrating that anesthesia can suppress Purkinje cell activity *in vivo* (Arancillo et al., 2015), and multiple reports of *mdx* Purkinje cell changes *in vitro* (Anderson et al., 2002; Pereira da Silva et al., 2018; Snow et al., 2014), we were next motivated to test whether cerebellar circuit dysfunction in *mdx* mice might be more robust and pronounced when analyzed in an awake and behaving context (Fig. 4).

Visual inspection of the raw traces shows more complex spikes in awake compared to anesthetized animals, as reported in previous studies (Arancillo et al., 2015). Upon further examination of the raw firing traces between *mdx* mice and controls, we determined that the structure of the spikes was similar between genotypes (Fig. 4C,D). Mean spike waveform shape was identical for both simple spikes and complex spikes between genotypes (Fig. 4E-H). In awake Purkinje cells, genotype had a significant effect on the set of simple spike mean firing rate, mean CV and mean CV2 [one-way MANOVA, Wilk's λ(1,46)=0.8266, *P*=0.0371]. Interestingly, mean simple spike firing rate was significantly different, at ∼19% lower in *mdx* Purkinje cells than in control cells (Fig. 4I; 69.1±3.4 vs 85.3±4.3 Hz; Wilcoxon rank sum test z=2.53, *P*=0.012). No effects were observed in the regularity of spikes (CV z=−0.03, *P*=0.98, CV2 z=−0.13, *P*=0.89). In addition, complex spikes also differed between genotypes in the awake state [Fig. 4J; Wilk's λ(1,46)= 0.7772, *P*=0.0106]. Although overall complex spike rate was not changed (Hz z=−0.55, *P*=0.58), overall complex spike variability was lower in *mdx* mice than in controls (CV=0.76±0.02 vs 0.92±0.04; z=3.17, *P*=0.0016). Complex spike CV2 was not affected in Purkinje cells that lack dystrophin (z=0.75, *P*=0.45). Together, these data demonstrate that, in awake animals, simple spike firing frequency is lower in *mdx* mice than in controls. It also reveals less variable complex spike firing in awake *mdx* mice compared to awake control mice.

We accounted for the expression of other dystrophin isoforms (i.e. Dp71, Dp116, Dp140 and Dp260) in the *mdx* Purkinje cells by probing for expression of the common C-terminal domain (Fig. S2). We confirmed antibody specificity by demonstrating heavy expression in the control hippocampus, as previously reported (Blake et al., 1999), and antibody localization to the plasma membrane of Purkinje cells (Fig. S2). We also evaluated the number of days since surgery (Fig. S3) and the recording depth [Fig. S4; evaluated with respect to dorsoventral and mediolateral coordinates relative to Paxinos and Franklin (2001) via labs.gaidi.ca/mouse-brain-atlas] as additional potential predictor variables. Through staining the C-terminal domain of the dystrophin protein, we found that the Purkinje cells of *mdx* mice have comparable dystrophin expression to control Purkinje cells (Fig. S2). This suggests that the *mdx* mutation and our electrophysiological findings are specific to the loss of the Dp427 isoform. To assess the impact of the days post-surgery and the recording depth on our data, we binarized each factor into low or high conditions relative to the median value (Figs S3 and S4), and found that neither factor had significant effects on awake simple spike firing in one-way MANOVAs [respective Wilk's λs: λ(1,46)=0.9613, λ(1,46)=0.9985; respective *P*-values: 0.63, 0.99]. We likewise examined the effect of predictor variables on complex spike firing in awake Purkinje cells. Again, the recording days post-surgery and recording depth did not show significant effects [λ(1,46)=0.8533, *P*=0.070; λ(1,46)=0.9943, *P*=0.97, respectively]. Moreover, because recording depth did not differ significantly between cells recorded in control compared to *mdx* mice (Wilcoxon rank sum test, z=0.58, *P*=0.56), it indicated that the differences in firing between genotypes were not attributable to differential sampling. These data suggest that, based on the factors we tested, the only factor showing a significant effect on simple spike and complex spike firing properties in awake mice was the genotype of the mice; namely, the loss of dystrophin, isoform Dp427, in the *mdx* mice. We therefore postulated that the simple spike and complex spike firing defects in the *mdx* mice should alter the ability of Purkinje cells to communicate their inhibitory output signals to their target neurons that are located in the three divisions of the cerebellar nuclei.

### Cerebellar nuclei output function is compromised in *mdx* mice

**Fig. 5.**
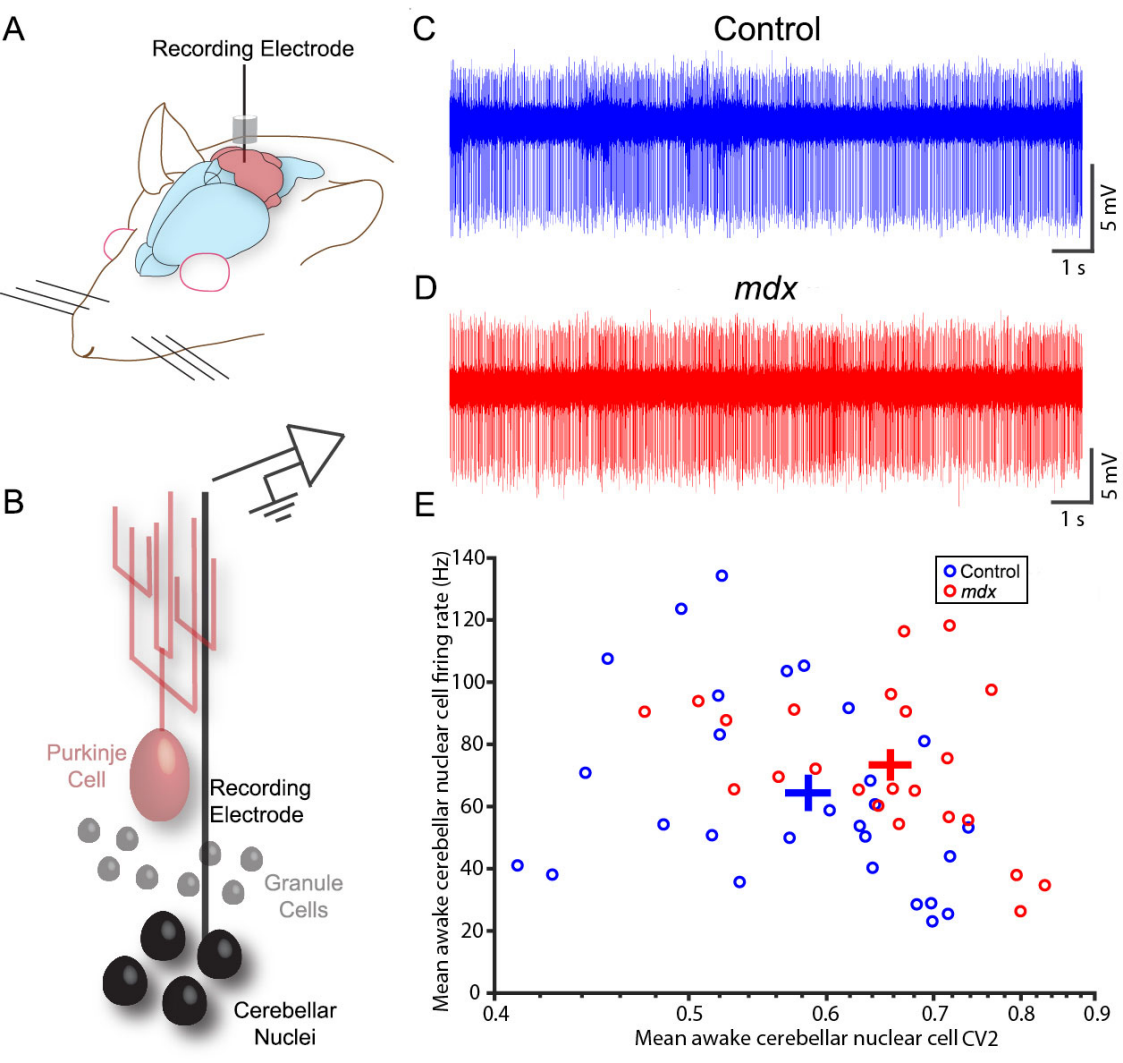
**Spontaneous firing properties of *mdx* and control cerebellar nuclear neurons in awake behaving mice.** (A) Schematic showing the recording approach. (B) Schematic illustrating the electrode placement for targeting the cerebellar nuclear neurons. (C) Example raw voltage trace from a control cerebellar nuclear neuron. (D) Example raw voltage trace from an *mdx* cerebellar nuclear neuron. (E) In awake mice, mean simple spike firing rate was not significantly different between the *mdx* mice (red) and controls (blue), but CV2 (variability) was higher in the *mdx* mice than in the controls. Circles are individual data points; crosses represent mean±s.e.m. *n*=28 control cells, *n*=23 *mdx* cells, *N*=4 mice of each genotype. Please refer to Table S1 for a list of the statistical tests used in this figure.

Having observed changes in Purkinje cell spike firing, we sought to determine what effect changes in their output would have on cerebellar nuclei firing (Fig. 5A,B). Raw voltage traces showed clear spike trains in the mutant mice (Fig. 5C,D). Days post-surgery, recorded position (dorsoventral, anteroposterior or mediolateral) and targeted nuclei all had no significant effect on firing properties in our recorded sample [λ(1,49)=0.9785, *P*=0.79; λ(1,49)=0.9022, *P*=0.18; λ(1,49)=0.9174, *P*=0.25; λ(1,49)=0.8891, *P*=0.13; λ(1,49)=0.9019, *P*=0.18, respectively; Fig. S5]. Genotype, however, did have a significant effect [λ(1,49)=0.7494, *P*=0.0033]. In pairwise testing, cerebellar nuclear firing rate and overall variability did not differ (z=−1.47, *P*=0.14, z=−0.71, *P*=0.48), but CV2 was ∼19% higher in *mdx* mice than in controls (Fig. 5E; CV2=0.46±0.02 vs 0.39±0.02; z=−2.36, *P*=0.018). This suggests that the net effect that lower Purkinje cell simple spike firing output and lower complex spike variability has on cerebellar nuclear cells in *mdx* mice is to increase the local variability of firing, or the variability in timing between one spike and the next. This effect is not likely due to a change in full-length dystrophin expression in the cerebellar nuclei themselves, because the very modest expression in a small population of cells is comparable between control and *mdx* mice (Fig. S6). It should be noted that, in many cells, the staining is barely beyond the level of background. Therefore, the changes we find in Purkinje cell activity appear to affect cerebellar nuclei output activity with little-to-no impact from dystrophin in the cerebellar nuclei. Activity changes in the cerebellar nuclei are predicted to alter the manner in which the cerebellum communicates with the rest of the brain and spinal cord during different behaviors.

## DISCUSSION

DMD causes progressive muscle degeneration and fibrosis, which leads to loss of motor strength and respiratory insufficiency (Falzarano et al., 2015). At later stages of disease, cardiomyopathy develops, with heart failure or respiratory complications leading to death, on average in the mid-30s, even with modern treatment regimens. Therapy for DMD has benefited significantly from studies of genetic mouse models of muscular dystrophy, including the *mdx* model developed in the late 1980s (Crone and Mah, 2018). Current investigational therapies for DMD with ongoing research in *DMD* mouse models include exon skipping, micro-dystrophin or surrogate gene therapy, gene editing, inflammation blockers and stem cell delivery (Long et al., 2016; Min et al., 2019). Canine models also offer immense insight into therapeutic approaches (Amoasii et al., 2018), although for studies of how DMD impacts brain anatomy and circuit function, the mouse still has advantages with respect to *in vivo* electrophysiology and complex genetics experiments. Therefore, in addition to their utility for measuring muscle pathology, DMD mice could be a promising avenue for uncovering the neural impairments often seen in individuals with DMD (Anderson et al., 2002; Rae and O'Malley, 2016; Vaillend et al., 2004). In this regard, the cerebellum has been a major target of interest (Cyrulnik and Hinton, 2008). Previous studies of *mdx* electrophysiology have reported changes in GABA release and uptake from cerebellar synaptosomes (Pereira da Silva et al., 2018), and lower firing rates and higher variability in the firing of dissociated Purkinje cells (Snow et al., 2014). However, whether these deficits are seen in awake mice has not previously been tested. Here, we tested this possibility and found that the loss of dystrophin alters Purkinje cell and cerebellar nuclei communication in behaving *mdx* mice.

The main goal of this paper was to investigate cerebellar function *in vivo*. The rationale was to determine whether the neurological defects reported in humans with DMD are reflected by changes in specific circuit functions in an alert state. Towards this, we recorded and examined the electrophysiological properties of cerebellar neurons in the intact brain, in an *mdx* mouse model of DMD. We chose to analyze only female mice to dissociate cerebellar functional output from potential compensation (Xu-Wilson et al., 2009; Zwergal et al., 2013) due to higher muscle degradation in males; however, it would be informative in future work to extend these analyses to the more severely affected male mice (Hakim and Duan, 2012; Hourdé et al., 2013; Salimena et al., 2000), or to heterozygous mice that express a relevant phenotype. Notably, we did not observe any overt behavioral deficits in the *mdx* mice at the ages we examined, suggesting that changes in the firing properties likely precede the onset of pathological motor symptoms.

Before interrogating circuit function, we first determined that several anatomical properties of the cerebellar microcircuit are unaffected in *mdx* mice, including cell type distribution, molecular layer thickness, molecular patterning and afferent terminal localization. We next recorded cerebellar activity *in vivo* and found a significant decrease of ∼19% in mean Purkinje cell simple spike firing rate in *mdx* mice compared to controls (Fig. 4). Two measures of simple spike variability showed no difference by genotype, but complex spikes were ∼17% less variable in the *mdx* mice compared to those in the controls (Fig. 4). The centrality of Purkinje cells in the cerebellar cortical circuits place these findings in a broader context of cerebellar function in disease (Reeber et al., 2013). Inputs from climbing fibers, inputs from mossy fiber–granule cell–parallel fiber pathways, and local modulation by inhibitory molecular layer interneurons all converge upon the Purkinje cell dendrites. Therefore, whatever the disease-causing injury (genetic or physical) or the ultimate impact on the circuit, the Purkinje cells are almost always involved in executing the response of the cerebellum. In mouse models of ataxia, *in vitro* electrophysiological analyses of Purkinje cell spiking reveal alterations as a consequence of molecular pathological defects and degeneration [SCA1 transgenic (Inoue et al., 2001)], which can start before the onset of obvious pathology [Purkinje cell-specific SCA2 transgenic (Hansen et al., 2013)], or due to changes in Purkinje cell intrinsic excitability [SK3-1B-GFP (Shakkottai et al., 2017)]. These data are supported by *in vivo* recordings demonstrating that Purkinje cell activity is dramatically altered in ataxia, as revealed in the *Car8^wdl^* model that exhibits a severe ataxia without neurodegeneration (Miterko et al., 2019a; White et al., 2016a). The significant tremor in *Car8^wdl^* also implicates the Purkinje cell firing defects as a source of the 4-12 Hz oscillations that might drive tremor pathophysiology (Kuo et al., 2019; White et al., 2016a). Purkinje cell firing defects are also the source of the high-stepping gait, abnormal twisting of the trunk and limbs, and poor mobility that is observed in different models of dystonia (Calderon et al., 2011; Fremont et al., 2015, 2017; Luna-Cancalon et al., 2014; White and Sillitoe, 2017). Here, we show that a mouse model of DMD, which is primarily a muscle degenerative disease but with clear neurological and neuropsychiatric components, contains cerebellar circuit deficits that fall into an expanding theme of activity-related firing disruptions. Defective Purkinje cell spiking could therefore mediate a wide range of behavioral changes in all conditions that involve cerebellar dysfunction.

Notably, Purkinje cells do not function in isolation: they synapse directly onto cerebellar nuclei neurons, which communicate cerebellar information to regions such as the thalamus, red nucleus, vestibular nuclei and the inferior olive. Because defects in Purkinje cell spike firing can have significant effects on their target neurons (Todorov et al., 2012; White et al., 2014), with resulting problems in motor control and learning (Schonewille et al., 2010; White et al., 2014), we also recorded from cerebellar nuclear neurons. Cerebellar nuclear neurons in *mdx* mice had a ∼19% higher local variability in firing (CV2) compared to those in control mice. Again, this result is reminiscent of the irregular cerebellar nuclei firing in ataxia (Hoebeek et al., 2008; White et al., 2014) and dystonia (Fremont et al., 2014; White and Sillitoe, 2017). However, it is unclear whether irregular firing of the cerebellar nuclei in one disease is exactly equivalent, at the behavioral level, to irregular firing in other diseases. Part of the complication is that the cerebellar nuclei have multiple targets, and it is unclear whether the same target neurons are implicated in different diseases. For example, it is postulated that the thalamic nuclei and connections to the basal ganglia are central to dystonia (Chen et al., 2014; White and Sillitoe, 2017), but is this the same pathway that also mediates motor defects in DMD? Perhaps, but DMD also involves non-motor dysfunctions, which are likely served through a different set of circuits. One argument is that the cerebellum might process all functions through a ‘universal cerebellar transform’ (Schmahmann et al., 2019). This theory postulates that regardless of whether the cerebellum is modulating action or cognition, its role as a coordinator of several neural tasks is the same. Certainly, there is strong clinical evidence for the cerebellum in emotion-based disorders (Schmahmann, 2019), and experimentally its contribution to behaviors such as reward and social behavior are emerging (Carta et al., 2019). Indeed, during non-motor function, the cerebellum must communicate with the cerebral cortex (Wagner et al., 2017; Wagner et al., 2019). Multiple anatomical pathways linking the cerebellum to cortical sites likely subserve these different non-motor functions (Bostan et al., 2013). The number of cerebral cortical sites that communicate with the cerebellum is potentially widely distributed (Marek et al., 2018), perhaps a prerequisite to the cerebellum's involvement in non-motor function in different and perhaps unexpected cases, such as DMD. It stands to reason that powerful cerebellar computations are necessary to drive such critical behaviors. One possibility is that the cerebellum promotes coherent activity between cortical brain regions; an example could be an adjustment in hippocampal–prefrontal cortex coherence upon Purkinje cell activation (McAfee et al., 2019). Interestingly, the hippocampus (Hoogland et al., 2019; Krasowska et al., 2014; Miranda et al., 2015, 2016) and prefrontal cortex (Suzuki et al., 2017) exhibit abnormalities in human DMD and *mdx* mice. It is therefore tantalizing to hypothesize that the decreased Purkinje cell simple spike output and the more variable cerebellar nuclear output in *mdx* mice could contribute to the motor or cognitive deficits reported in individuals with DMD.

Previous reports have noted changes in the distribution of GABA_A_ receptors on Purkinje cells in *mdx* mice (Grady et al., 2006). Although this molecular change could potentially contribute to the altered firing we report in *mdx* Purkinje cells, it is currently not clear what effect it might have. Specifically, because Purkinje cell dendrites have active rather than passive conduction properties, the exact distribution of GABA_A_ receptors is critical to predicting their effect on Purkinje cell output. For instance, in biophysical simulations, distal dendritic inhibition has a larger effect on firing rate than proximal inhibition (Solinas et al., 2006). Therefore, it is difficult to predict how the changes in *mdx* GABA_A_ receptor distribution would ultimately impact Purkinje cell firing properties without a more detailed and quantitative description of the exact composition of dendritic channels in the *mdx* mice, and across the different cerebellar lobules. We also note that dystrophin is expressed in at least seven isoforms (Muntoni et al., 2003), and while the full-length Dp427 isoform is the one most strongly expressed in Purkinje cells (Górecki et al., 1992), smaller isoforms like Dp71 and Dp140 could potentially compensate to some degree for the *mdx* mutation and loss of Dp427. Nevertheless, our results point to an important role for Dp427 in cerebellar function that cannot completely be compensated for by other isoforms.

The loss of dystrophin expression in *mdx* mice does not prevent specific cell populations from differentiating, eliminate certain classes of neurons or prevent any of the main cerebellar cell types from attaining their proper position within the tri-laminar cerebellar cortex (Fig. 1). However, despite the correct location of cells and afferent fibers, cerebellar zonal topography is altered. The cerebellum is organized into a series of sagittal zones that are defined by neuronal birth date, lineage restriction, embryonic gene expression, afferent topography, neuronal spike properties and molecular marker expression (Apps et al., 2018; Miterko et al., 2019b). Zones are organized around the Purkinje cells, which express the most striking of all zonal patterns. Previous work demonstrated that dystrophin is not expressed at equal levels in all Purkinje cells, but instead in a pattern of zones that fall within the fundamental cerebellar map (Blake et al., 1999). In both the muscle and brain, dystrophin binds to a complex of proteins including the dystrobrevins and a coiled-coil protein called dysbindin (Benson et al., 2001). Dysbindin is heavily expressed in mossy fiber terminals, where it also forms an array of zones that respect the topography of Purkinje cells (Sillitoe et al., 2003). Loss of dystrophin in *mdx* mice alters the terminal field distribution of dysbindin-expressing mossy fibers (Sillitoe et al., 2003). The changes in Purkinje cell function and the accompanied defects in mossy fiber patterning in the *mdx* model is reminiscent of ataxic mice that have a lack of Purkinje cell neurotransmission with poorly defined mossy fiber zones (White et al., 2014). Moreover, there is no overt neurodegeneration in either model. It therefore is tempting to speculate that the behavioral deficits in *mdx* mice are, at least in part, due to zonal miswiring. Based on the distribution of firing properties, we likely recorded from Purkinje cells in zebrinII+ as well as zebrinII− zones (Figs 3 and 4) (Xiao et al., 2014; Zhou et al., 2014). If there are functional zonal defects in *mdx* mice, we predict their involvement in a number of different behaviors. However, how motor and non-motor information are simultaneously encoded in zones is unclear, although the convergence of Purkinje cell zonal information within the cerebellar nuclei (Person and Raman, 2011) would have an impact on how signals are altered in *mdx* mice (Fig. 5). Specifically, changes in complex spikes would be predicted to alter cerebellar output (Tang et al., 2019), especially since the climbing fiber projections also adhere strictly to the boundaries of the Purkinje cell zonal map (Gravel et al., 1987; Sugihara and Quy, 2007) and contribute to behavior (Horn et al., 2010). Within each zone, loss of dystrophin could, in theory, impact learning mechanisms via zone-dependent changes in neurotransmission and plasticity (Kostadinov et al., 2019; Paukert et al., 2010; Wadiche and Jahr, 2005). Similar to the synchronous activation of cell ensembles that control individual muscles (Welsh et al., 1995), cerebellar firing properties and circuit patterning could also instruct non-motor communication across higher-order brain centers. Our extracellular recordings included cells in lobules VI-VII of the vermis and CrusI and CrusII of the hemispheres, regions known to be involved in motor behaviors, with additional and growing evidence that they contribute to various non-motor behaviors (Koziol et al., 2014; McAfee et al., 2019; Shipman and Green, 2019). Based on the abnormalities we uncovered, we speculate that the function of cerebellar dystrophin complexes could mediate such functions *in vivo*, during behavior.

### Conclusion

DMD is a debilitating disease that results in death. Boys are affected in the severe forms, which start with muscle degeneration, although cardiac and respiratory problems often determine the endpoint of the disease. Unfortunately, during the course of disease, afflicted individuals also have to deal with neurological deficits that may be motor as well as non-motor in nature. A number of brain regions, such as the hippocampus and prefrontal cortex, could mediate the cognitive defects in humans and in animal models such as the *mdx* genetic model. In addition, the cerebellum, a region involved in motor and non-motor functions, has been implicated in DMD. Behavioral studies as well as *in vitro* electrophysiology experiments in *mdx* mice support a contribution of the cerebellum to DMD. Here, we used *mdx* mice to show that Purkinje cells fire abnormally *in vivo*, and that their altered spiking properties are communicated downstream to cerebellar nuclear neurons, which are also defective. We demonstrate that cerebellar output is more variable, a circuit modification that could have signaling consequences similar to diseases such as ataxia, tremor and dystonia. We postulate that erroneous cerebellar output could mediate the motor and non-motor abnormalities in DMD.

## MATERIALS AND METHODS

### Animal maintenance

Mouse husbandry and experiments were performed under an approved Institutional Animal Care and Use Committee (IACUC) protocol at Baylor College of Medicine. Female C57BL/10ScSnJ control (stock #000476; *N*=22) and C57BL/10ScSn-*Dmd^mdx^*/J homozygous mutant (stock #001801; *N*=22; *mdx/mdx*) mice were obtained from The Jackson Laboratories (Bar Harbor, ME, USA). The use of this specific control mouse line is based on known difficulties in breeding the mutant strain and is supported by previous work demonstrating the validity of their use (Rybalka et al., 2014; Xu et al., 2019; Yoon et al., 2019). We used only one sex to avoid any potential sex differences in cerebellar function (Mercer et al., 2016). Specifically, we chose to use female mice to try to dissociate the effect of the loss of dystrophin as much as possible from potential compensatory changes in cerebellar output; changes in muscle function could potentially induce compensatory changes in cerebellar activity (Xu-Wilson et al., 2009; Zwergal et al., 2013), and female mutants have more modest muscular deficits than male mutants when aged less than 6 months (Hakim and Duan, 2012; Hourdé et al., 2013; Salimena et al., 2000). Of note, though, our previous work using female homozygous (*mdx/mdx*) and male hemizygous (*mdx/Y*) mice did not reveal differences in cerebellar functional anatomy or sensitivity to molecular patterning changes between the sexes (Sillitoe et al., 2003). Regardless, in this work, we analyzed cerebellar function in adult, 2- to 5-month-old female mice.

### Surgery

For anesthetized recordings, the mice were anesthetized with 3% isoflurane and then with a cocktail containing 75 mg/kg ketamine and 0.5 mg/kg dexmedetomidine. We then transferred the mice from the anesthesia chamber to a stereotaxic platform (David Kopf Instruments, Tujunga, CA, USA) that is integrated with an *in vivo* recording rig. Throughout the experiment, mice were connected to a breathing tube and isoflurane concentration was maintained at 0.15-0.25%. The head was fixed in place with metal ear bars. Fur in the back of the head was removed using depilatory cream. An anteroposterior incision was made with a scalpel blade to expose the skull. Depending on the age, the muscle was cut and reflected away if the area for the craniotomy was covered. All craniotomies were performed 0-1.5 mm to the right of the midline with reference to bregma (Paxinos and Franklin, 2001), and above the region that approximately corresponds to lobules VI-VII of the vermis or CrusI and CrusII of the hemispheres. A hole ∼1 mm in diameter was drilled into the skull using a Dremel handheld rotary drill (model 4000). Once the skull was drilled to translucence, the remaining bone and cartilage were etched with an 18-gauge needle until a circular flap could be removed to expose the brain. The craniotomy was performed carefully because even minimal tissue damage causes brain and blood vessel pulsations, which inhibit stable single-unit recordings. Moisture within the craniotomy was maintained by keeping the opening full with drops of 0.9% w/v saline solution. During anesthesia, Purkinje cells were recorded between 0.2 mm and 2.4 mm (median=1.1 mm) ventral to the surface of the brain.

For awake recordings, surgical procedures were similar to those we previously reported (White et al., 2016b; Stay et al., 2019), with the following modifications: mice were anesthetized with 3% isoflurane at induction, followed by 1-2% isoflurane for maintenance. After the craniotomy, a custom 2-mm circular plastic recording chamber was secured to the skull with dental acrylic, along with a metal head plate for restraint. A small metal post was implanted over bregma for stereotaxic reference during recordings. After surgery, the mice were transferred to a warmed box chamber until they returned to sternal recumbency, at which point they were returned to their home cages to continue recovering. Recordings began after three or more days post-surgery recovery. Mice were head-fixed on a foam running wheel, where they were free to make limb or whisking movements, though higher speeds of walking or running were inhibited by a high coefficient of friction of wheel rotation. Purkinje cells were recorded 5.5-7.7 mm (median=6.8 mm) posterior and 0.5-2.0 mm (median=1.1 mm) lateral to bregma, at 0.3-2.9 mm (median=1.7 mm) deep from the surface of the brain. Cerebellar nuclei were targeted between 1.8 mm and 4.0 mm (median=2.8 mm) ventral from the brain surface, and below surface coordinates of 5.8-7.6 mm (median=6.2 mm) posterior and 0.5-2.0 mm (median=1.4 mm) lateral to bregma. The precise stereotaxic coordinates relative to bregma were used to reconstruct the recording sites post hoc.

### Electrophysiology: spike properties and data analysis

Single-unit recordings were attained with 2-5 MΩ Tungsten electrodes (Thomas Recording, Germany) that are controlled by a motorized micromanipulator (MP-225, Sutter Instrument). The signals were band-pass filtered at 0.3-13 kHz, amplified with an ELC-03XS (NPI, Tamm, Germany) amplifier, and digitized into Spike2 (CED, Cambridge, UK). Well-isolated units were analyzed over 200-300 s in anesthetized animals (median=200 s), and 40-120 s in awake animals (median=71 s). The anesthetized mice were secured in a stereotaxic unit during the recordings, whereas the awake animals were recorded during quiet wakefulness while on a foam wheel (White et al., 2016b). Analysis of the raw traces was performed with Spike2, Excel (Microsoft) and MATLAB (MathWorks). Purkinje cells were identified by the presence of simple spikes and complex spikes. Simple spikes are generated intrinsically within Purkinje cells and are modulated by mossy fiber to granule cell afferent inputs, whereas complex spikes are a Purkinje cell-specific action potential that result exclusively from the excitatory climbing fiber input. Complex spikes cause a subsequent pause of ∼20 ms in simple spike firing. Simple spikes and complex spikes were sorted independently. Cerebellar nuclear neurons were identified by their significant and characteristic depth within the cerebellum, as well as their general firing profile that typically sounds distinct from Purkinje cells when isolated with the aid of an audio amplifier. Based on the stereotaxic coordinates that were used for targeting the electrodes, we predict that most cerebellar nuclear neurons were recorded from the fastigial and interposed nuclei, with some potentially isolated from deeper within the vestibular nuclei. The spike trains for both cell types were analyzed for frequency (Hz=spikes/s). To quantify the average variability in their firing pattern, the interspike interval (ISI) CV [(standard deviation of ISIs)/(mean of ISIs)] was calculated. To measure the variability in firing patterns within a short period of two interspike intervals, the CV2 [CV2=2*|ISI_n+1_−ISI_n_|/(ISI_n+1_+ISI_n_)] was calculated (Holt et al., 1996). Throughout the study, numerical results are reported as mean±s.e.m. Pairwise statistical comparisons of the different firing properties were performed with the non-parametric Wilcoxon rank sum test (Table S1). Significance was set at α=0.05 threshold. Sample size was not determined using *a priori* power analysis, but was based on the statistical criteria for significance in observations. Animals were not randomized into analysis groups, and no animals or samples were excluded. Data are available upon request.

### Statistics of electrophysiological recordings

We examined several different predictor variables and their relationship to several response variables. Specifically, we tested the null hypotheses that the predictor variables [genotype, days post-surgery at recording and recorded depth (also indicates the putative lobule)] had no significant effects on the means of the response variables (mean firing rate, mean CV and mean CV2). We tested these hypotheses through an initial omnibus MANOVA test for each predictor variable. If the MANOVA result showed likelihood of *P*<0.05 that the means of each response variable were equal between the levels of the predictor variable, we rejected the null hypothesis and proceeded with pairwise comparisons to determine which response variable(s) differed. Since we did not know the true underlying variance levels for each response variable, we used non-parametric Wilcoxon rank sum tests rather than Student's *t*-tests, to avoid assuming equal variance. The significance levels of all statistical tests were corrected for multiple comparisons with the Benjamini–Hochberg correction. A *P*-value less than the Benjamini–Hochberg critical value with false discovery rate set to 0.2 was interpreted to reject the null hypothesis of equal means in a given response variable between the predictor groups. Please refer to Table S1 for a list of the statistical tests used in this study.

### Tissue preparation and cutting

For perfusion-fixation, animals were deeply anesthetized with avertin (2,2,2-tribromoethanol), and then perfused through the heart with 0.1 M phosphate-buffered saline (PBS; pH 7.2), followed by 4% paraformaldehyde (PFA) diluted in PBS. After incubation in 4% PFA overnight, the tissue was placed into 70% ethanol before embedding in wax for paraffin tissue processing (Sillitoe et al., 2008). Paraffin tissue sections were cut at 10 µm on a microtome and ribbons collected on positively charged glass slides from a warm water bath. Alternatively, for free-floating tissue processing, the brains were post-fixed in 4% PFA for 24-48 h after perfusion, cryoprotected stepwise in sucrose solutions (15% and 30% diluted in PBS), embedded in Tissue-Tek OCT Compound (Sakura Finetek, Torrence, CA, USA), and then frozen at −70°. The tissue was cut at 40 µm on a cryostat and collected into 24-well plates.

### Immunohistochemistry

Immunohistochemistry was carried out as described previously (Reeber and Sillitoe, 2011; Sillitoe et al., 2003, 2010; White and Sillitoe, 2013). Briefly, tissue sections were thoroughly washed, blocked with 10% normal goat serum (NGS; Sigma-Aldrich, St Louis, MO, USA) for 1 h at room temperature and then incubated in 0.1 M PBS containing 10% NGS, 0.1% Tween-20 and primary antibodies for 16-18 h at room temperature, shaking gently. The tissue sections were then washed three times in PBS and incubated in goat anti-mouse horseradish peroxidase (HRP)-conjugated secondary antibodies (1:200; DAKO, Carpinteria, CA, USA) for 2 h at room temperature, again shaking gently. The cerebellar tissue was subsequently rinsed, and immunoreactivity was analyzed using SG substrate (Vector Laboratories, Burlingame, CA, USA) as a chromogen. After mounting the tissue onto glass slides, sections were coverslipped using Cytoseal mounting medium (Richard-Allen Scientific, San Diego, CA, USA). We tested the specificity of the secondary antibodies by processing the tissue sections without primary antibodies. No signal was detected in such control experiments, indicating that the staining we observed was not due to non-specific signals from the HRP antibodies (data not shown). Please refer to our previous work for additional tests of reliable signals from the antibodies used in this study (Reeber and Sillitoe, 2011; White and Sillitoe, 2017; White et al., 2014, 2016b).

### Antibodies

Anti-dystrophin antibody (1:20, NCL-DYS1), purchased from Leica Biosystems (Newcastle upon Tyne, UK), was used to verify that full-length dystrophin was eliminated in *mdx* mice. To account for the expression of the other dystrophin isoforms (i.e. Dp71, Dp116, Dp140 and Dp260) in control and *mdx* Purkinje cells, we used an anti-dystrophin (MANDRA1) antibody (1:100; NB120-7164) from Novus Biologicals (Centennial, CO, USA), targeted to the C-terminus. Mouse monoclonal anti-calbindin-D28K (calbindin; 1:10,000; CD38), rabbit polyclonal anti-parvalbumin (1:1000; PV25) and rabbit polyclonal anti-calretinin (1:500; CR7699/3H) were purchased from Swant (Marly, Switzerland). Calbindin specifically labeled Purkinje cells, parvalbumin labeled Purkinje cells and GABAergic interneurons, and calretinin labeled unipolar brush cells. We also used anti-CAR8 primary antibodies (1:3000; Santa Cruz Biotechnology, Santa Cruz, CA, USA) to label Purkinje cells (Miterko et al., 2019a). To assess excitatory synapses, we used mouse monoclonal anti-vesicular glutamate transporter 2 (VGLUT2; 1:500; MAB5504), purchased from Chemicon (Millipore, Billerica, MA, USA), for visualizing climbing and mossy fiber terminals (Gebre et al., 2012; Hisano et al., 2002; Reeber and Sillitoe, 2011), and rabbit polyclonal anti-vesicular glutamate transporter 1 (VGLUT1; 1:500; 135 302), purchased from Synaptic Systems (Goettingen, Germany), for visualizing parallel fiber synapses and mossy fiber terminals (Gebre et al., 2012). The granule cell layer was assessed by using a granule cell marker, rabbit anti-GABARα6, which was purchased from Millipore (1:500; AB5610). Mouse monoclonal anti-NFH (also called anti-SMI-32; 1:1500) was purchased from Covance (Princeton, NJ, USA). Anti-SMI-32 recognizes the non-phosphorylated form of NFH (see manufacturer product datasheet for details), which on tissue sections labels the soma, dendrites and axons of adult Purkinje cells, and also basket cell axons and pinceaux (Demilly et al., 2011). Rabbit anti-neurogranin (1:500) was raised against full-length recombinant rat neurogranin protein (Chemicon, Temecula, CA, USA; AB5620). Neurogranin recognizes Purkinje cells in the perinatal cerebellum and Golgi cells in the adult cerebellum (Larouche et al., 2006; Singec et al., 2003). Rabbit polyclonal anti-cocaine- and amphetamine-related transcript peptide (CART 55-102; H-003-62) was used at a concentration of 1:250 to detect climbing fibers (Reeber and Sillitoe, 2011) and was purchased from Phoenix Pharmaceuticals (Burlingame, CA, USA). To fully visualize the fibers, the CART signal was amplified using a biotinylated secondary antibody and the Vectastain Elite ABC method from VectorLabs (Burlingame, CA, USA; Reeber and Sillitoe, 2011).

### Fluorescent Nissl histology

For dual staining of calbindin and NeuroTrace, 10 µm or 40 µm tissue sections were immunolabeled with primary antibody as described above (calbindin; 1:10,000; CD38). We then added NeuroTrace fluorescent Nissl stain (Molecular Probes, Eugene, OR, USA), diluted to 1:1000, directly to the secondary antibody cocktail. Alexa-Fluor^®^-conjugated secondary antibodies (1:1500; Life Technologies; A31570) were used to amplify and detect the calbindin signal. After three PBS rinses, we mounted the stained tissue sections with FLUORO-GEL mounting medium (Electron Microscopy Sciences, Hatfield, PA, USA) onto glass slides before imaging.

### Imaging and data analysis

Photomicrographs of tissue sections were captured with a Zeiss AxioCam MRc5 camera mounted on a Zeiss Axio Imager.M2 microscope. Images of tissue sections were acquired with the Zeiss Apotome.2 system and analyzed using Zeiss ZEN software (2012 edition). After imaging, the raw data were imported into Adobe Photoshop CS5 and corrected for brightness and contrast levels. Schematics were drawn in Adobe Illustrator CS5.

### Quantification of anatomical features

Cerebellar size, molecular layer thickness, Purkinje cell number, and cerebellar nuclei areas and densities were quantified from three control and three *mdx* mice using ImageJ software (https://imagej.nih.gov/ij/). Cerebellar size and Purkinje cell numbers were determined by calculating, then averaging, the areas or the soma numbers of three to eight sections of tissue, per animal, respectively. All of the cerebellar tissue used in these calculations was previously stained with calbindin and/or Nissl and was taken from within 200 µm from the midline. We used the tissue area, inclusive of all ten vermal lobules, measured from sagittal sections, as a proxy for examining cerebellar size. The number of Purkinje cells was calculated for the whole sagittal cerebellum (lobules I-X), the anterior zone of the cerebellum (lobules I-V), the central zone of the cerebellum (lobules VI-VIII) and the posterior/nodular zones of the cerebellum (lobules IX-X). To complement our quantification of Purkinje cell number, molecular layer thickness and cerebellar nuclei areas and densities were additionally measured. Molecular layer thickness was measured in regions of cerebellar cortex flanking the fissure between lobules III and IV, on sagittal sections that were co-stained with calbindin and Nissl. We chose to quantify these specific regions because the cerebellar cortex in these lobules has substantial stretches without curves, which makes quantification more systematic when comparing between mice. For each mouse, the molecular layer thickness measurements were averaged from four to eight sections separated by ∼200 μm around the midline. For additional details on the quantification of molecular layer thickness, see Brown et al. (2019). Finally, we quantified the areas and densities of the fastigial and interposed cerebellar nuclei in three control and three *mdx* mice, each using three sagittal tissue sections stained with calbindin (the Purkinje cell axons in the cerebellar nuclei provide an ideal outline for assessing general nuclei anatomy) and Nissl. The fastigial nuclei measurements were calculated from sagittal tissue sections that were ±0.48 μm to ±1.08 μm from the midline, whereas the interposed nuclei measurements were calculated from sagittal tissue sections that were ±1.08 μm to ±1.92 μm from the midline. We did not quantify the area of the dentate nuclei because our recordings in this study are predicted to be from the fastigial and interposed cerebellar nuclei. Nuclear densities were determined in ImageJ software by first converting the TIFF images containing the cerebellar nuclei to a 16-bit resolution, then adjusting the threshold so that the Nissl staining of the nuclei was accurately detected by the ‘Analyze Particles’ plug-in. The subsequent nuclear counts were then divided by 50 μm to obtain the average number of cell nuclei present in any given 50-μm area. For all of the anatomical features quantified, numerical results are reported as the mean±s.e.m. Sample size was determined based on the statistical criteria for significance in observations. As previous reports have described morphological features of cells in the cerebellum as being well approximated by normal distributions (Ristanović et al., 2011), pairwise comparisons of the quantifications of these anatomical features were performed with two-tailed Student's *t*-tests.

This article is part of a special collection ‘A Guide to Using Neuromuscular Disease Models for Basic and Preclinical Studies’, which was launched in a dedicated issue guest edited by Annemieke Aartsma-Rus, Maaike van Putten and James Dowling. See related articles in this collection at http://dmm.biologists.org/collection/neuromuscular.

## Supplementary Material

Supplementary information
