# Supplementary material for: In vivo cerebellar circuit function is disrupted in an mdx mouse model of Duchenne muscular dystrophy
Source: Dis Model Mech. 2019 Dec 9;13(2):dmm040840. doi: 10.1242/dmm.040840 (PMC6906634; doi:10.1242/dmm.040840)
Supplement: Supplementary information [file dmm-13-040840-s1.pdf]

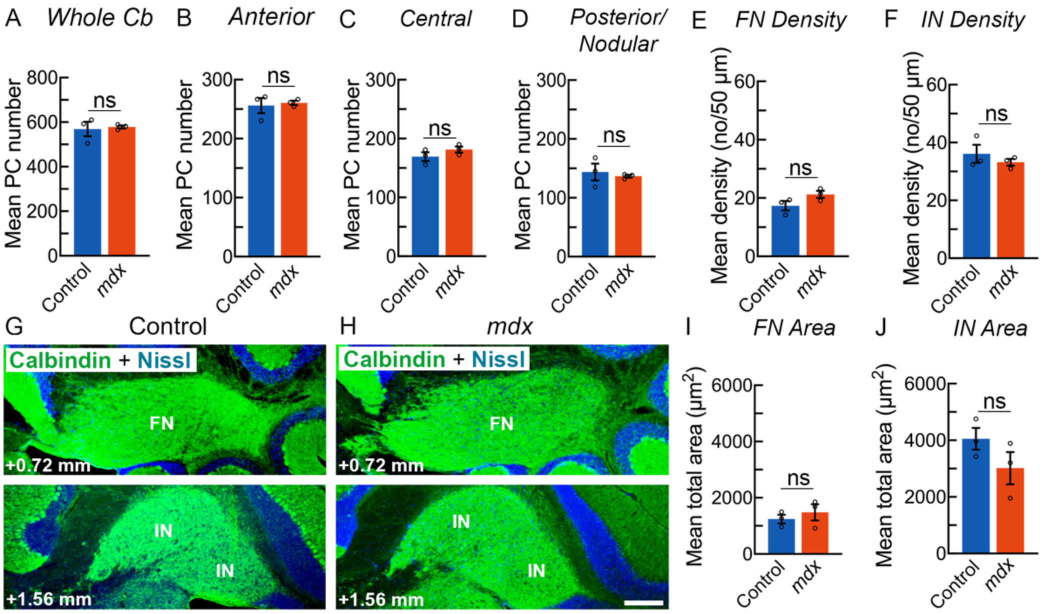

Fig. S1. The anatomy of cerebellar circuits is grossly intact in *mdx* mice. A. The total number of Purkinje cells (PC) in the whole *mdx* cerebellum (Cb) does not differ statistically from the number of Purkinje cells (PC) in the whole control cerebellum (counted in all lobules/per sagittal section; Control = 569.2 ± 32.7, *mdx*= 578.7 ± 6.7, two-tailed Student's t-tests, N = 3 animals per genotype, n = 3-8 sections per animal, t(4) = -0.286, p = 0.79). **B-D.** The total number of Purkinje cells (PC) in the *mdx* cerebellum does not differ from that of controls when analyzed by zone (Control = 256.0 ± 12.9 (anterior), 169. ± 7.4 (central), 143.8 ± 14.3 (posterior/nodular), *mdx*= 260.6 ± 3.6 (anterior), 181. ± 5.1 (central), 136.7 ± 2.3 (posterior/nodular), two-tailed Student's t-tests, N = 3 animals per genotype, n = 3-8 sections per animal; t(4) = -0.340, p = 0.75, t(4) = -1.345, p = 0.25, t(4) = 0.489, p = 0.65, respectively). **E-F**. The fastigial (FN) and interposed (IN) nuclear densities (no = number) are comparable between the genotypes (Control = 17.36 ± 1.61 (FN), 36.11 ± 3.12 (IN), *mdx* = 21.26 ± 1.20 (FN), 33.19 ± 1.14 (IN), two-tailed Student's t-tests, N = 3 animals per genotype, n = 3 sections per animal; t(4) = -1.9646, p = 0.12, t(4) = 0.880, p = 0.43, respectively). **G-J.** The areas of the fastigial (FN) and interposed (IN) cerebellar nuclei are comparable between control and *mdx* mice (Control = 1,242.5 ± 154.9 µm^2 (FN), 4,051.9 ± 385.4 µm^2 (IN), *mdx* = 1,483.5 ± 287.7 µm^2 (FN), 3,015.8 ± 567.9 µm^2 (IN), two-tailed Student's t-tests, N = 3 animals per genotype, n = 3 sections per animal, t(4) = -0.737, p = 0.50, t(4) = 1.510, p = 0.21, respectively). The distances of the fastigial and interposed nuclei from bregma are +0.72 mm (FN) and +1.56 mm (IN), respectively. Calbindin staining (green) labels Purkinje cell soma, axons and terminals whereas Nissl (blue) is a general stain for cell nuclei. Scale bar, 50 µm.

Disease Models & Mechanisms • Supplementary information

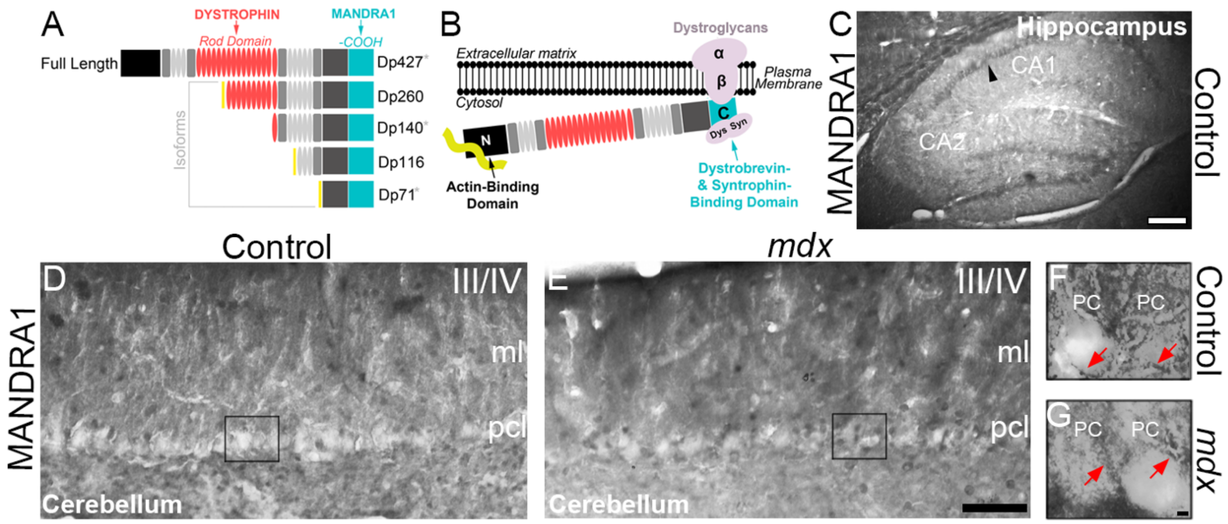

Fig. S2. Purkinje cells in control and *mdx* mice express multiple isoforms of dystrophin. A. Schematic detailing the different isoforms of dystrophin. Gray asterisks indicate the isoforms that are expressed in the mammalian brain. The dystrophin antibody used in **Fig. 1** targets the rod domain (red) while the MANDRA1 antibody used in this supplemental figure targets the c-terminus (teal), which is present in all of the isoforms. **B.** The MANDRA1 antibody detects dystrophin isoforms that are located near the plasma membrane since the c-terminus of the dystrophin protein interacts with glycoproteins embedded in the plasma membrane. **C.** The hippocampus expresses MANDRA1 in the control brain, demonstrating the expected pattern of expression. **D-E.** MANDRA1 expression in control (**D**) and *mdx* (**E**) Purkinje cells is identical. Representative examples of Purkinje cells are outlined. **F.** Examples of control Purkinje cells (PC) that express MANDRA1 (red arrows). **G.** Examples of *mdx* Purkinje cells (PC) that express MANDRA1 (red arrows). Scale bar (**C**), 200 µm. Scale bar (**D-E**), 50 µm. Scale bar (**F-G**), 10 µm. Molecular layer (ml). Purkinje cell layer (pcl). N = 3 mice per genotype.

Disease Models & Mechanisms • Supplementary information

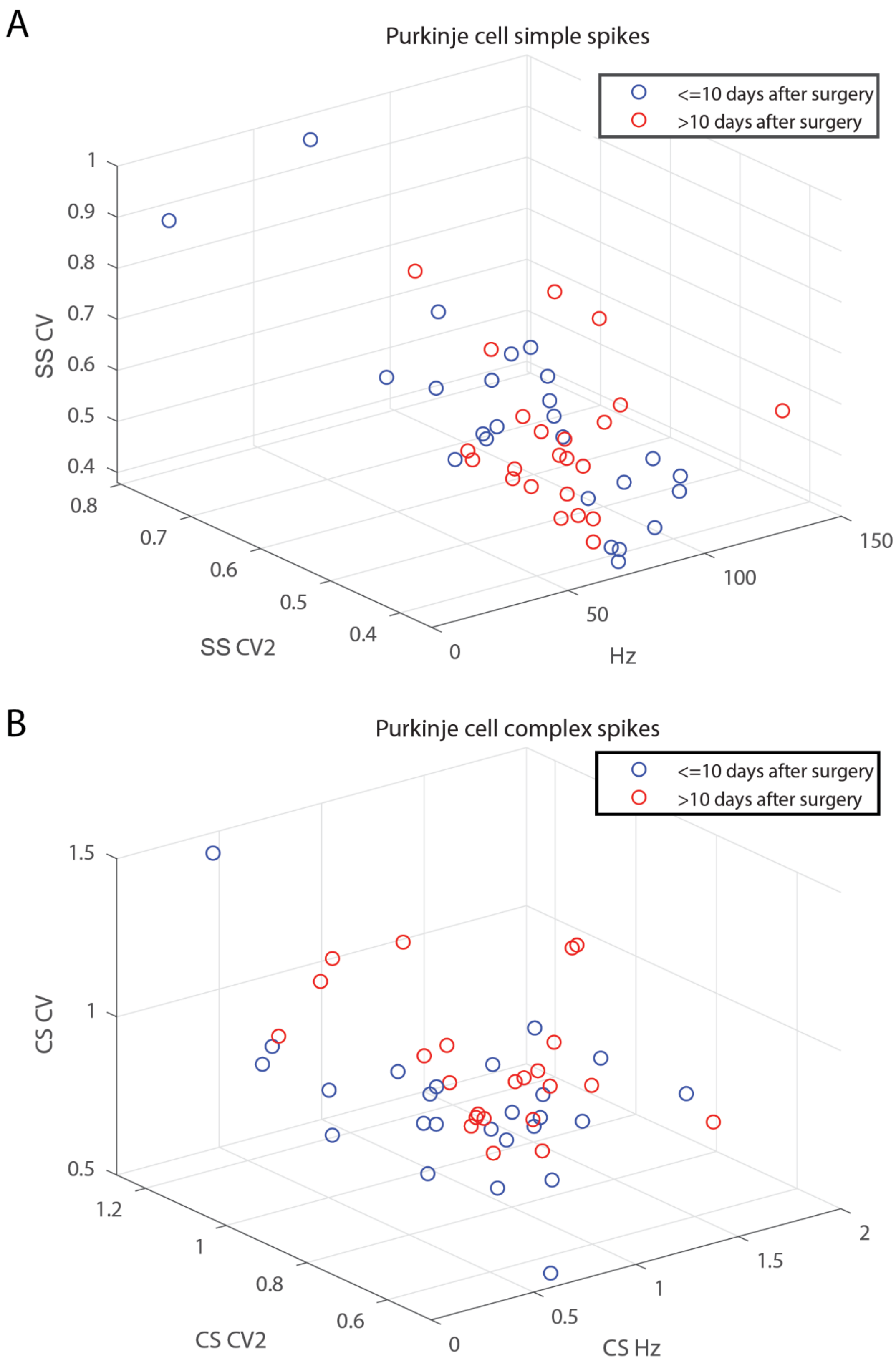

Disease Models & Mechanisms • Supplementary information

Fig. S3. The time between surgery and recording does not affect the Purkinje cell properties. The session day following surgery in which analyzed awake Purkinje cells were recorded did not affect simple spike (**A**) or complex spike (**B**) summary statistics when comparing neurons recorded 10 days or fewer following surgery (n = 25) versus those recorded more than 10 days after surgery (n = 23). For days post-surgery as a predictor variable, recorded cells were divided into two groups, high and low, both relative to the median value. The median value was 10, but with two cells recorded exactly 10 days after surgery. Please refer to **Table S1** for a list of the statistical tests used in this figure.

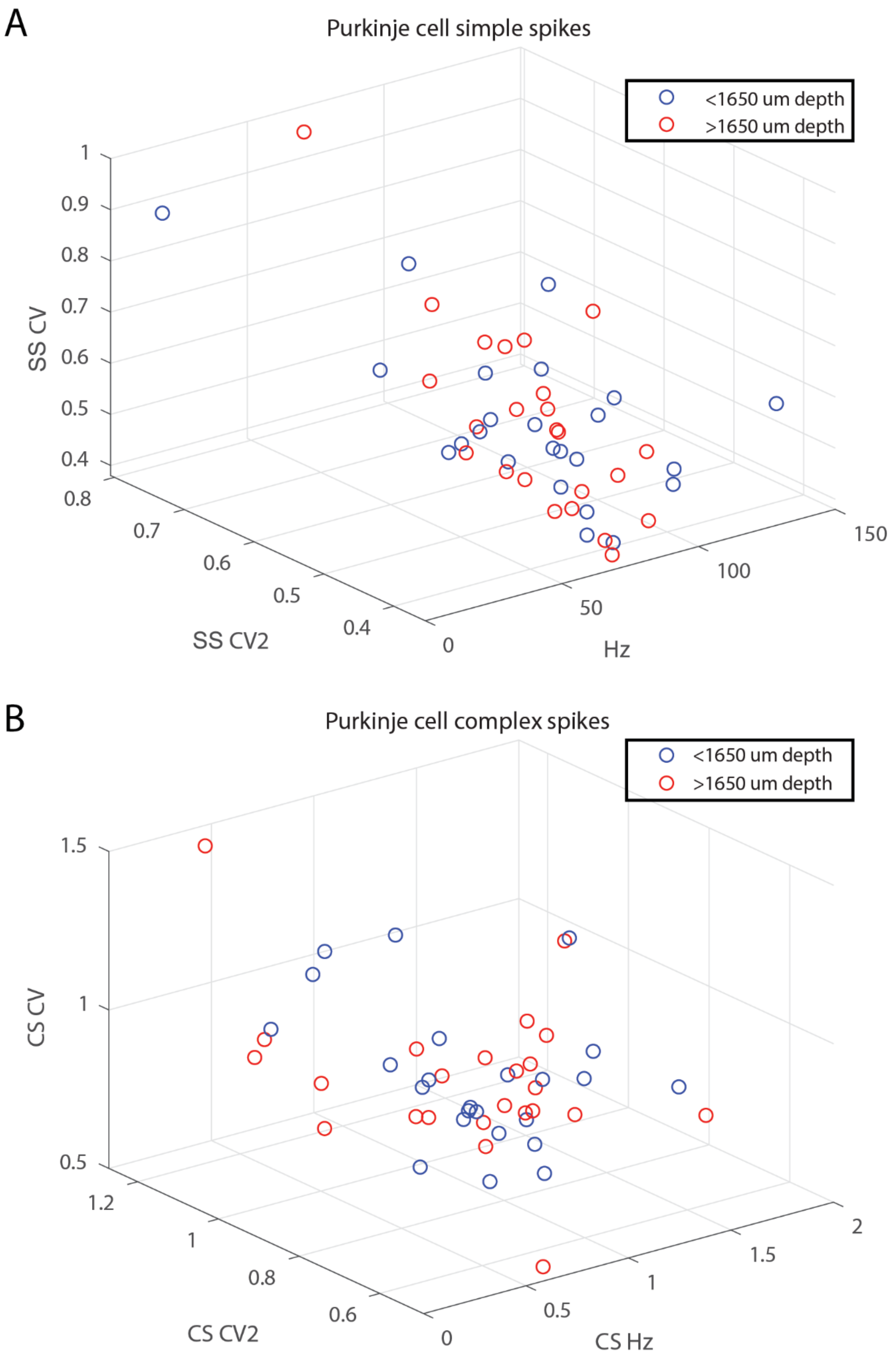
A
Purkinje cell simple spikes
<1650 um depth
>1650 um depth
SS CV
1
0.9
0.8
0.7
0.6
0.5
0.4
SS CV2
0.8
0.7
0.6
0.5
0.4
Hz
0
50
100
150
B
Purkinje cell complex spikes
<1650 um depth
>1650 um depth
CS CV
1.5
1
0.5
CS CV2
1.2
1
0.8
0.6
CS Hz
0
0.5
1
1.5
2

Fig. S4. The depth of the recordings does not affect overall Purkinje cell properties. The depth of recorded awake Purkinje cells did not affect simple spike (**A**) or complex spike (**B**) summary statistics when comparing neurons recorded dorsal to 1650 µm (n = 24) and those recorded ventral to 1650 µm (n = 24). The depth of recorded neurons was calculated based on where we found the recording features at optimal signal to noise ratio with respect to the surface of the cerebellum at the site of the craniotomy. The recorded cells were recorded into two groups, high and low, relative to the median value. Therefore, as a population the dorsoventral depth indicates neurons that fall in the upper half versus the lower half of the analyzed distance. Please refer to **Table S1** for a list of the statistical tests used in this figure.

Disease Models & Mechanisms • Supplementary information

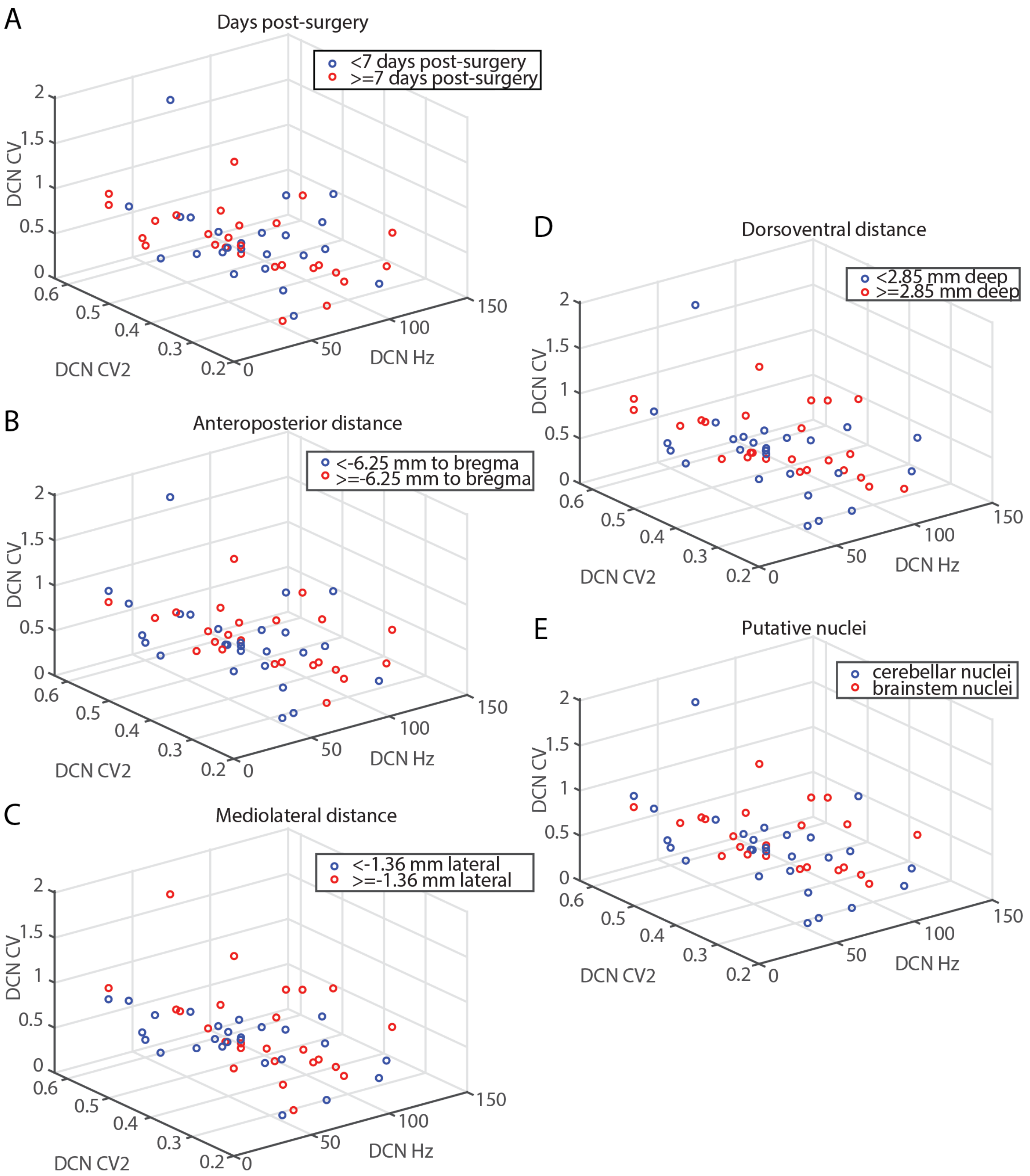

Disease Models & Mechanisms • Supplementary information

Fig. S5. Potential predictor variables apart from genotype show no effect on spiking characteristics in cerebellar nuclear neurons. A. The length of time between craniotomy surgery and recording date did not significantly affect rate, CV, or CV2 (one-way MANOVA p = 0.79). **B.** Anteroposterior recording position showed no difference in spiking statistics between the anterior and posterior half of recorded cells (p = 0.25). **C.** Mediolateral distance was not a significant factor influencing cerebellar nuclear firing (p = 0.13). **D.** Dorsoventral recording depth did not affect spiking statistics (p = 0.18). **E.** Putative recorded nuclei showed no significant effect on spiking statistics (p = 0.18).

Disease Models & Mechanisms • Supplementary information

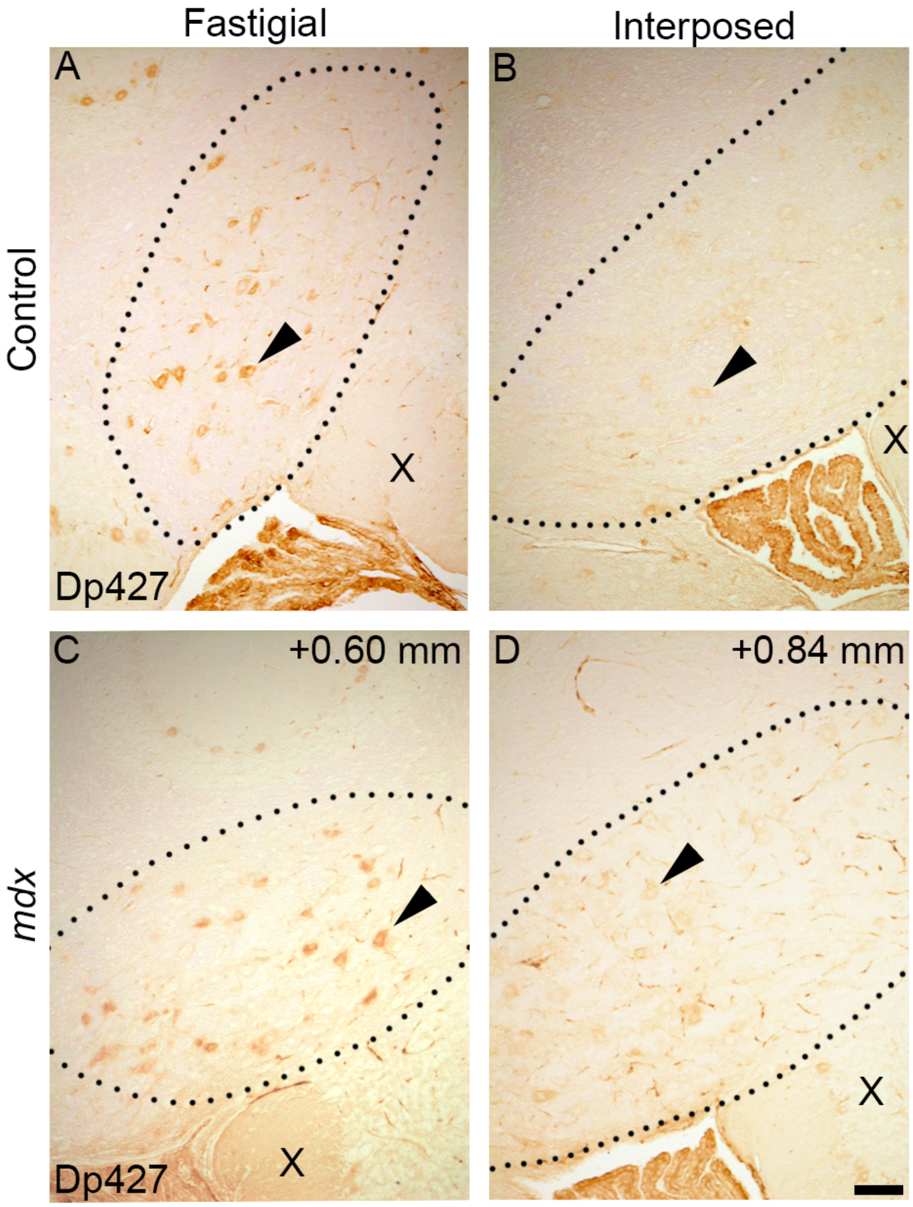

Fig. S6. Dystrophin is weakly expressed in a subset of cells in the cerebellar nuclei of control and *mdx* mice. A-B. A small number of cells in the fastigial (**A**) and interposed (**B**) cerebellar nuclei are weakly immunoreactive for the Dp427 isoform in control mice. **C-D**. Anti-dystrophin staining in the fastigial (**C**) and interposed (**D**) cerebellar nuclei of *mdx* mice reveals an identical staining pattern to that of control mice. The expression of Dp427 is weaker in specific regions of the interposed nuclei of both genotypes. In many cells within both cerebellar nuclei, the expression of Dp427 is barely beyond the level of background staining. Scale bar (**A-D**), 200 µm. N = 3 mice per genotype.

Comparison	Statistical Test	N	Statistic	Degrees of freedom	Likelihood	BH-corrected significance
Overall cerebellar area by genotype	Two-tailed t-test	6	t = 0.280	4	p = 0.7936	0
Molecular layer thickness by genotype	Two-tailed t-test	6	t = -0.623	4	p = 0.5668	0
Purkinje cell number by genotype, whole cerebellum	Two-tailed t-test	6	t = -0.286	4	p = 0.7888	0
Purkinje cell number by genotype, anterior zone	Two-tailed t-test	6	t = -0.3401	4	p = 0.7509	0
Purkinje cell number by genotype, central zone	Two-tailed t-test	6	t = -1.3452	4	p = 0.2498	0
Purkinje cell number by genotype, posterior-nodular zones	Two-tailed t-test	6	t = 0.4894	4	p = 0.6502	0
Fastigial nucleus area by genotype	Two-tailed t-test	6	t = -0.7372	4	p = 0.5019	0
Fastigial nucleus density by genotype	Two-tailed t-test	6	t = -1.9457	4	p = 0.1236	0
Interposed nucleus area by genotype	Two-tailed t-test	6	t = 1.5096	4	p = 0.2056	0
Interposed nucleus density by genotype	Two-tailed t-test	6	t = 0.8796	4	p = 0.4287	0
Anesthetized simple spike response variables by genotype	One-way MANOVA	94	λ = 0.9028	1,92	p = 0.0261	1
Anesthetized simple spike firing rate by genotype	Wilcoxon rank sum	48/46	z = 1.99	93	p = 0.0471	0
Anesthetized simple spike CV by genotype	Wilcoxon rank sum	48/46	z = 1.76	93	p = 0.0787	0
Anesthetized simple spike CV2 by genotype	Wilcoxon rank sum	48/46	z = -1.48	93	p = 0.1392	0
Anesthetized complex spike response variables by genotype	One-way MANOVA	94	λ = 0.9929	1,92	p = 0.8859	0
Awake simple spike response variables by genotype	One-way MANOVA	48	λ = 0.8266	1,46	p = 0.0371	1
Awake simple spike firing rate by genotype	Wilcoxon rank sum	24/24	z = 2.53	47	p = 0.0115	1
Awake simple spike CV by genotype	Wilcoxon rank sum	24/24	z = -0.03	47	p = 0.9753	0

Awaked simple spike CV2 by genotype	Wilcoxon rank sum	24/24	z = -0.13	47	p = 0.8934	0
Awake complex spike response variables by genotype	One-way MANOVA	48	λ = 0.7772	1,46	p = 0.0106	1
Awake complex spike firing rate by genotype	Wilcoxon rank sum	24/24	z = -0.55	47	p = 0.5848	0
Awake complex spike CV by genotype	Wilcoxon rank sum	24/24	z = 3.17	47	p = 0.0016	1
Awake complex spike CV2 by genotype	Wilcoxon rank sum	24/24	z = 0.75	47	p = 0.4517	0
Awake CN response variables by genotype	One-way MANOVA	51	λ = 0.7494	1,49	p = 0.0033	1
Awake CN firing rate by genotype	Wilcoxon rank sum	28/23	z = -1.47	50	p = 0.1424	0
Awake CN CV by genotype	Wilcoxon rank sum	28/23	z = -0.71	50	p = 0.4778	0
Awake CN CV2 by genotype	Wilcoxon rank sum	28/23	z = -2.36	50	p = 0.0184	1
Awake simple spike response variables by days	One-way MANOVA	48	λ = 0.9613	1,46	p = 0.6250	0
Awake simple spike response variables by dorso-ventral position	One-way MANOVA	48	λ = 0.9943	1,46	p = 0.9954	0
Awake complex spike response variables by days	One-way MANOVA	48	λ = 0.8533	1,46	p = 0.0701	0
Awake complex spike response variables by dorso-ventral position	One-way MANOVA	48	λ = 0.9943	1,46	p = 0.9682	0
Recording depth by genotype	Wilcoxon rank sum	24/24	z = 0.58	47	p = 0.56	0
Awake CN response variables by days post-surgery	One-way MANOVA	51	λ = 0.9785	1,49	p = 0.7933	0
Awake CN response variables by anterior-posterior position	One-way MANOVA	51	λ = 0.9174	1,49	p = 0.2515	0
Awake CN response variables by medio-lateral	One-way MANOVA	51	λ = 0.8891	1,49	p = 0.1337	0
Awake CN response variables by dorso-ventral	One-way MANOVA	51	λ = 0.9022	1,49	p = 0.1800	0
Awake CN response variables by putative lobule	One-way MANOVA	51	λ = 0.9019	1,49	p = 0.1791	0

Table S1. Post-hoc statistical analyses employed throughout the study. A tabulated version of the data showing the values that were reported for the post-hoc statistical analyses.
